# Fully Printed Ionic Diodes from Polymerizable Ionic Liquids

**DOI:** 10.1021/acsnano.6c06135

**Published:** 2026-07-27

**Authors:** Alexander Q. Kane, Eric Yang, Ryan L. Truby

**Affiliations:** † Department of Materials Science and Engineering, 3270 Northwestern University, Evanston, Illinois 60208, United States; ‡ Department of Mechanical Engineering, Northwestern University, Evanston, Illinois 60208, United States

**Keywords:** 3D printing, ionic diodes, iontronics, poly(ionic liquids), rectification

## Abstract

Soft ionic diodes (SIDs) comprising p- and n-type polyelectrolytes are fundamental components of bioinspired iontronic devices. However, traditional ionic diode fabrication techniques require low-throughput, manual processes to interface electrodes and electrolytes and are typically limited to producing planar geometries. Moreover, hydrogels commonly used as SID electrolytes lose water over time, which leads to device instability and loss of performance. In this work, we present a method for fully 3D printing ionic diodes from solvent-free, polymerizable ionic liquids (pILs) that enables rapid and scalable manufacturing of SIDs. Four inks, including p- and n-type electrode and electrolyte inks, are used to print SIDs with 2D to 3D geometries and rectification ratios of up to 46. The geometry-, scan rate-, and time-dependent performance of SIDs are characterized via cyclic voltammetry and chronoamperometry. Leveraging the flexibility of 3D printing, we demonstrate current and voltage scaling relationships in multidiode arrays printed in parallel or series, respectively. We also highlight a dense multidiode crossbar array (≈100 SIDs/cm^2^) that can be quickly and uniquely achieved with our 3D printing approach. We anticipate the materials and methods reported in this work will enable opportunities for fabricating ionic diodes and multifunctional iontronic devices critical to advancing applications in bioelectronics, neuromorphics, and soft robotics.

## Graphical abstract

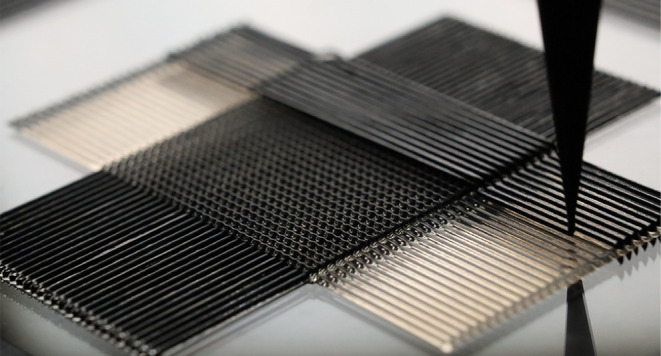

By using ions instead of electrons as charge and information carriers, soft, solid-state iontronic devices have emerged with unique advantages for bioinspired sensing,^1–4^ bioelectronic,^5–8^ neuromorphic,^9,10^ and robotic applications.^11–13^ Iontronic devices require the integration of multiple soft ionic and electronic conductors within architectures that enable the on-demand formation and selective manipulation of ionic gradients. Soft ionic diodes (SIDs) are a fundamental building block in iontronics that rectify ionic currents and prevent diffusion across concentration gradients of multiple ionic conductors.^14–16^ Despite being single components, SIDs have demonstrated capabilities beyond traditional rectification, such as synaptic plasticity in ionic circuits,^17,18^ multimodal sensing,^19,20^ and even reversible electrostatic clutching.^21,22^ Compared to soft electronics, iontronics opens opportunities for multifunctional behaviors with comparably simple devices that are often low-voltage, energy-efficient, and biocompatible.^23,24^ However, realizing the broader potential of SID-based iontronic devices requires manufacturing processes capable of rapidly integrating all requisite ionic and electronic conductors in arbitrary forms to achieve the complex, heterogeneous, 3D architectures that such devices will require.

SIDs are typically constructed from pairs of poly­(anionic) and poly­(cationic) electrolytes as p- and n-type networks with mobile cations and anions, respectively. Mobile ions diffuse across the polyelectrolyte heterojunction when the interface is formed, establishing an ionic double layer (IDL) that functions as a capacitive barrier. As in semiconductor diodes, applied electric fields modulate the strength of the IDL, allowing or preventing the flow of mobile ions under forward and reverse bias.^25–27^ Hydrogels are the most common polyelectrolytes used in SIDs and solid-state iontronic devices.^28–33^ p- and n-type hydrogels are typically cast into thin films or molded into microscale features and then assembled into SIDs. However, their susceptibility to water loss, even under ambient environmental conditions, presents challenges for long-term SID operation in iontronic devices. Furthermore, integrating solid electrodes with aqueous/gel electrolytes, often through multiple processing and assembly steps, restricts SIDs to 2D planar form factors. Manufacturing processes like multimaterial 3D printing of ionic conductors and dielectric materials are increasingly used to produce soft iontronic sensors and electroluminescent devices with heterogeneous 3D forms that enable new capabilities not possible with planar designs.^34–36^ Recent efforts have extended extrusion-based 3D printing to the fabrication of hydrogel SIDs through microgel processing,^37^ surfactant-supported assembly of microscale hydrogels,^38^ and photopolymerizable hydrogels.^39^ Still, additive manufacturing methods that can yield geometrically complex, microscale SIDs and fully integrated iontronic devices,^40^ comprising materials more robust than hydrogels and complete with all necessary electrode and polyelectrolyte features, have not yet been reported.

In this work, we present a method for fully 3D printing SIDs from polymerizable ionic liquids (pILs) via multimaterial direct-ink writing (DIW) (Figure 1). Nonaqueous, solvent-free pIL electrolytes have recently emerged as a promising alternative to hydrogels and are particularly attractive for SIDs because their low volatility and high charge density permit rectification without a loss in conductivity or change in mechanical properties over time.^16,41^ We formulated four extrudable, IL monomer-based inks for printing p- and n-type pIL electrolytes and their corresponding electrodes. Our process minimizes blending at the heterojunction and prevents interfacial failure between materials. We demonstrate multimaterial assembly of pIL SIDs across a range of 3D structures and characterize their structural-, scan-rate-, and history-dependent performance. Finally, we showcase printed assemblies and dense microscale arrays of pIL SIDs to highlight the opportunities opened through rapid 3D printing of ionic diodes.

**Figure 1:**
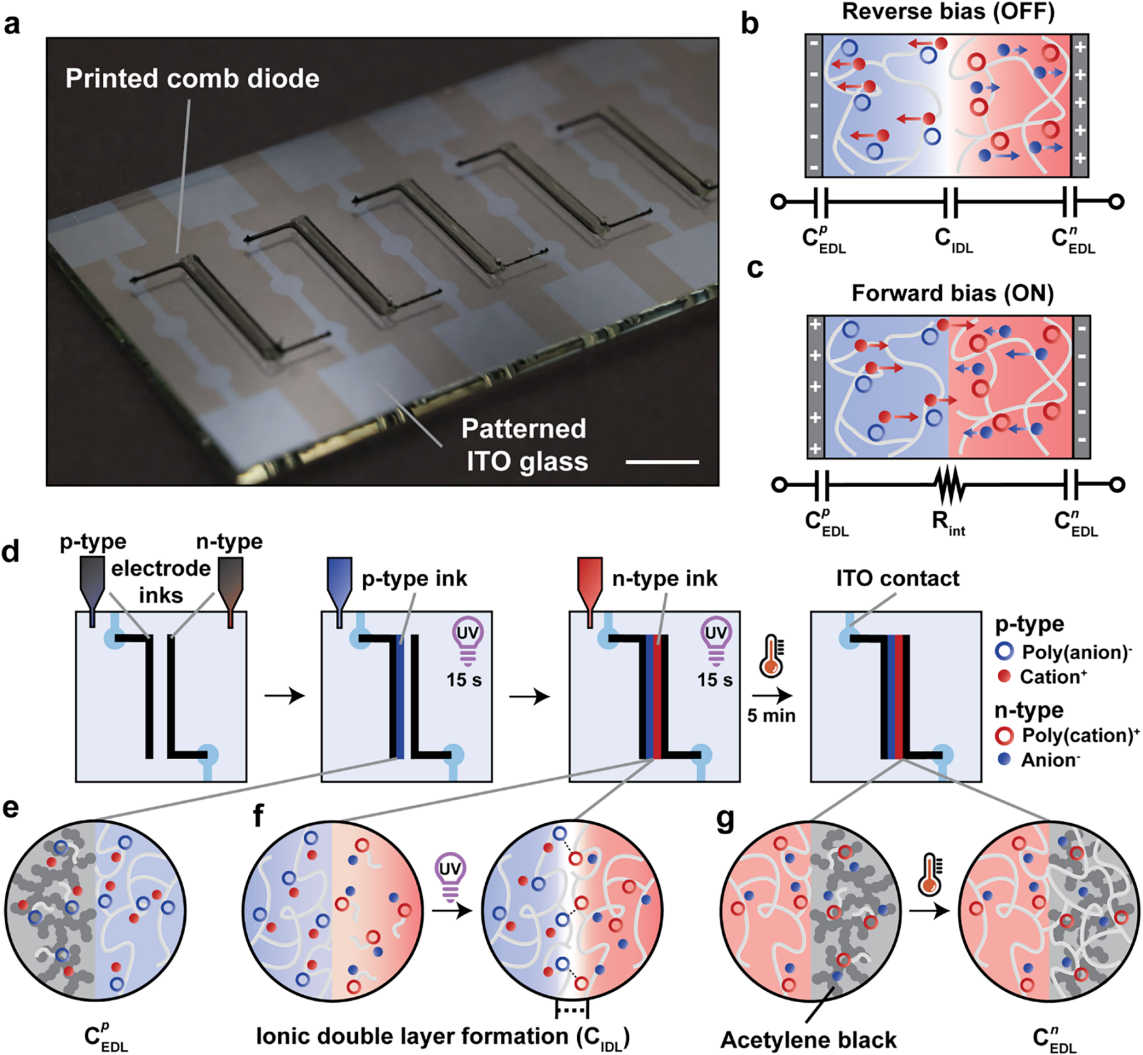
Multimaterial 3D Printing of pIL SIDs. (a) A photograph shows five ppnn comb SIDs printed on a glass substrate with patterned ITO electrodes. Scale bar is 5 mm. (b) Under a reverse (negative) bias, mobile ions migrate toward the electrodes, reinforcing the IDL and restricting current. (c) Under a forward (positive) bias, mobile charges migrate toward the interface, destroying the IDL and permitting current. (d) The schematic illustrates how two pairs of p-/n-type electrode and electrolyte inks are printed to form a SID. (e) The same IL monomer is the base for p-/n-type electrode and electrolyte inks. (f) An IDL forms at the interface of photopolymerized p-/n-type electrolyte inks. (g) After printing, electrode inks are thermally cured to create a coherent interface with electrolyte inks.

## Results and Discussion

### Printing pIL SIDs

SIDs rectify ionic currents by controlling charge transport across a heterojunction interface.^16,25–27^ In pIL heterojunctions, an IDL forms when p- and n-type polyelectrolytes are brought into contact. Mobile cations and anions, driven by competing thermodynamic and entropic processes, rearrange near the heterojunction interface to create a region that is depleted of mobile ions. The separation of mobile ions at the IDL creates a capacitive boundary with capacitance ,*C*_IDL_, and a nonzero, built-in open-circuit potential, *V*_OCP_, at rest. Applying a negative potential (i.e., a reverse bias) across the SID depletes the IDL of mobile ions and restricts current flow across the device (Figure 1b), while an applied positive potential (i.e., a forward bias) drives mobile ions toward the IDL, permitting current and replacing the capacitive IDL (*C*_IDL_) with a resistive interface (*R*_int_, Figure 1c). In addition to electrical inputs, current flow can be induced across an SID with mechanical deformation via the piezoionic effect.^16,24^ Electric double layers (EDLs) form at all electrode-electrolyte interfaces. Therefore, manufacturing processes that produce SIDs must preserve all of the interfaces that give rise to their function.

To print SIDs with multimaterial DIW,^42,43^ we require four inks: a pair of inks to print p- and n-type polyelectrolytes to form the heterojunction, and a pair of inks to print electrodes that interface with each polyelectrolyte. The central material selection for printing SIDs, and therefore our ink designs, is the polyelectrolytes that form the heterojunction. Hydrogels are the gold standard polyelectrolyte for SIDs given their ease of use and wide commercial availability,^44–46^ but, as we show in Figure S1, dehydration leads to the failure of hydrogel SIDs over time. Nonvolatile electrolytes like pILs overcome the challenges presented by dehydration, which are especially important for microscale SIDs. The highly tunable synthesis and chemical properties of pIL electrolytes are also appealing, as they open opportunities to a broad range of mobile ions and mechanical properties for iontronic applications.^47–51^ For our polyelectrolyte inks, we select IL monomers that have demonstrated rectification comparable to hydrogels in manually assembled pIL SIDs^16,41,52^ and are amenable to 3D printing.^34,53–55^ To fully print SIDs, the library of extrudable soft electrode inks^56^ is limited to conductive additives that are electrochemically compatible with pIL electrolytes to avoid redox reactions. Carbon-based electrodes are inert to most electrolytes and can be readily interfaced with pIL electrolytes by creating composite inks comprising IL monomers and carbon nanoparticles, such as acetylene black.

Any process for 3D printing SIDs must also overcome the fabrication limitations of manual assembly. Manually assembled SIDs are not intact. Typically, SID electrolytes are not bonded with electrodes and require mechanical clamping,^16,22^ while p-n electrolyte interfaces are held together by weak electrostatic interactions. Our strategy uses covalent crosslinking at all interfaces to maintain a strong interfacial bond between all materials. To aid covalent crosslinking at electrode-electrolyte interfaces, we use two electrode inks laden with the same p- or n-type IL monomers used in the p-/n-type electrolyte inks, respectively. However, crosslinking at the p-n electrolyte interface must avoid mixing p-/n-type IL monomers to maintain a functional heterojunction. Mixing can arise from the diffusion of IL monomers before polymerization or from shear during extrusion of adjacent materials. We circumvent this challenge by designing UV-curable electrolyte inks that can be polymerized mid-process.

Figure 1d illustrates the process for printing a comb SID, the simplest SID structure we explore in this work. First, the p- and n-type electrode inks are printed onto a glass substrate with pre-patterned ITO electrodes. The electrode inks contain acetylene black nanoparticles that impart electrical conductivity and their corresponding p- and n-type IL monomer (Figure 1e). A p-type electrolyte ink is then deposited adjacent to the p-type electrode and photocrosslinked under UV light for 15 s to form a poly­(anionic) network with mobile cations. The electrode inks do not cure with UV exposure due to the high UV absorption of the acetylene black nanoparticles (Figure 1e). Next, the n-type electrolyte ink is printed between the p-type electrolyte and the n-type electrode and immediately photocrosslinked for 15 s. During UV exposure, the n-type electrolyte ink forms a poly­(cationic) network with mobile anions, which leads to the creation of the p-n electrolyte interface (Figure 1f). Photocrosslinking the printed p-type electrolyte before printing the n-type electrolyte ink is essential for minimizing diffusion and/or mixing of the two IL monomers at the heterojunction, which, as we show in Figure S2 for various print process variations, otherwise leads to poor SID rectification. After printing, the SID is thermally cured at 80^◦^C for 5 min under an inert atmosphere to crosslink the p-type/n-type electrode inks (Figure 1g) and form a complete comb SID. The entire process for fabricating a single comb SID takes place in less than 10 min; fabrication time decreases when multiple SIDs are printed at once (see Figure 1a and Video S1).

Successful printing depends on rheologically tailored polyelectrolyte and electrode inks, which we formulated from the IL monomers shown in Figures 2a,b. The p-type IL monomer, 1-ethyl-3-methylimidazolium (3-sulfopropyl)­acrylate (ES), and the n-type IL monomer, 1-[2-acryloyloxyethyl]-3-butylimidazolium bis­(trifluoromethane)­sulfonimide (AT), are synthesized in-house following modified literature procedures (see *Experimental Section*). The neat IL monomers are viscous, Newtonian liquids (Figure S3). Following previous works that have shown ILs can be loaded with nanoparticles to develop extrudable, paste-like fluids^57,58^ the electrode inks are formulated by mixing their respective IL monomer with 14 wt % acetylene black nanoparticles, 0.4 mol % crosslinker (poly­(ethylene glycol) diacrylate, PEGDA), and 0.4 mol % low-temperature thermal initiator (2,2’-azobis­(4-methoxy-2,4-dimethylvaleronitrile) V-70). The acetylene black nanoparticles impart both electrical conductivity (Figure S4) and shear-thinning behavior to the electrode inks for extrusion-based 3D printing. The V-70 thermal initiator is stable for up to ≈12 h at room temperature but rapidly cures in less than 5 min at 80^◦^C. The electrolyte inks are formed by mixing their respective IL monomer with 9 wt % (p-type) or 6 wt % fumed silica nanoparticles (n-type), 0.5 wt % crosslinker (PEGDA), and 0.5 wt % photoinitiator (liquid ethyl (2,4,6-trimethylbenzoyl)-phenylphosphinate, TPO-L). Rheological characterization reveals that all inks are shear-thinning (Figure 2c) and yield-stress fluids (Figure 2d). The apparent viscosities, viscoelastic moduli, and yield stresses of the electrode inks are at least an order of magnitude higher than those of the electrolyte inks. As shown in Figure 2d, the plateau storage moduli $\left(G_{0}^{\prime }\right)$ of the p-type electrode, n-type electrode, p-type electrolyte, and n-type electrolyte inks are 210, 410, 20, and 13 kPa, respectively. Their respective shear yield stresses, τ_*y*_, are 910, 1700, 31, and 90 Pa. The electrolyte inks are intentionally soft yield-stress fluids to maximize the weight fraction of IL monomers while still supporting extrusion of self-supporting features and layer-by-layer deposition. Similarly, the electrode inks are designed to maximize the weight fraction of acetylene black nanoparticles without clogging a 200 μm nozzle. Stiff electrode inks also prevent shear-induced mixing during extrusion of electrolytes. The formulation of these four extrudable inks enables multimaterial DIW of SIDs.

**Figure 2:**
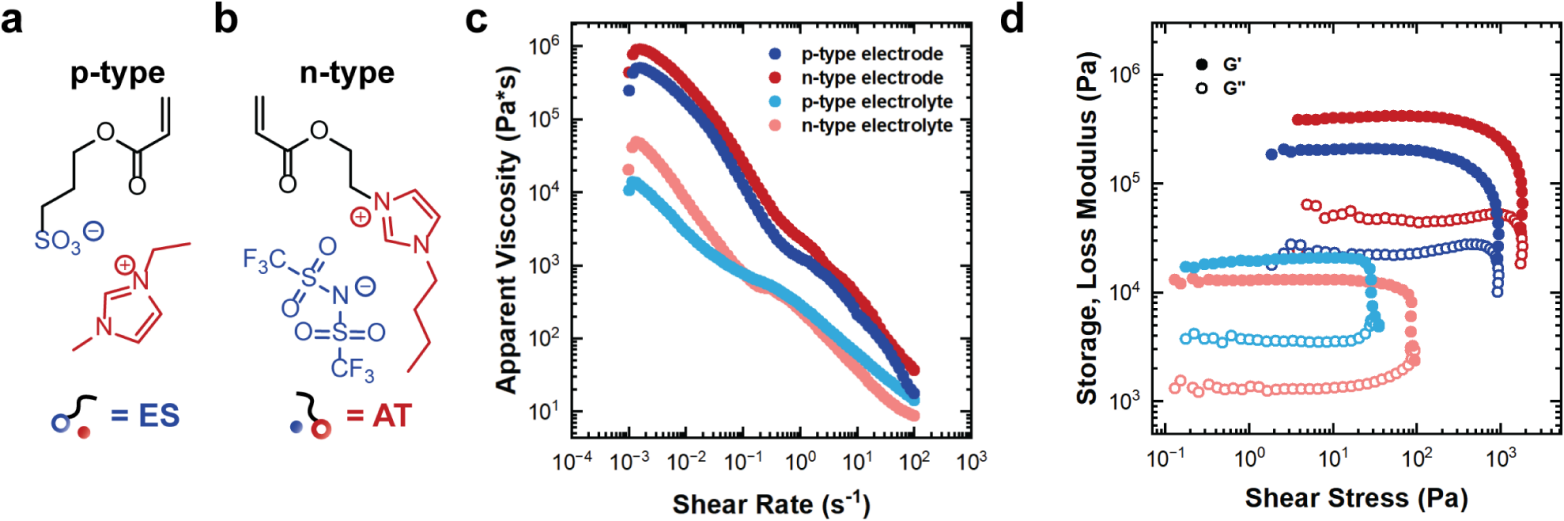
Inks for Printing pIL SIDs. Molecular structures for (a) p-type (ES) and (b) n-type (AT) IL monomers used in electrode and electrolyte inks. Plots of (c) apparent viscosity versus shear rate and (d) storage (G’) and loss (G”) moduli versus shear stress for the electrode and electrolyte inks.

### Electrochemical Characteristics of Printed pIL SIDs

To understand how printed SIDs perform, we printed SIDs with three architectures: a 2D comb SID (Figure 3a), a 2.5D spiral SID (Figure 3b), and a 3D stack SID (Figure 3c). Each SID geometry is printed following the same key steps for heterojunction formation, as detailed in Figure 1. The three geometries utilize similar print parameters (e.g., print speed and extrusion pressure) but are fine-tuned depending on the structural needs. For example, the electrolyte inks in the spiral SID are slightly overextruded compared to the comb SID to encase electrodes and prevent them from short circuiting. Similarly, for stack SIDs, the n-type electrolyte ink is overextruded to minimize air bubbles at the heterojunction. SIDs are printed on glass substrates patterned with ITO features as electrodes. ITO electrodes prevent stray current from deforming during electrical contact and characterization.^16^ To evaluate the influence of printed electrodes on SID performance, we also printed comb, spiral, and stack SIDs with only electrolyte inks. For the remainder of the paper, we denote SIDs printed without and with the electrode inks as pn and ppnn SIDs, respectively. We use different glass substrates with pn- and ppnn-specific ITO features when printing SIDs with and without electrode inks (Figure S5).

**Figure 3:**
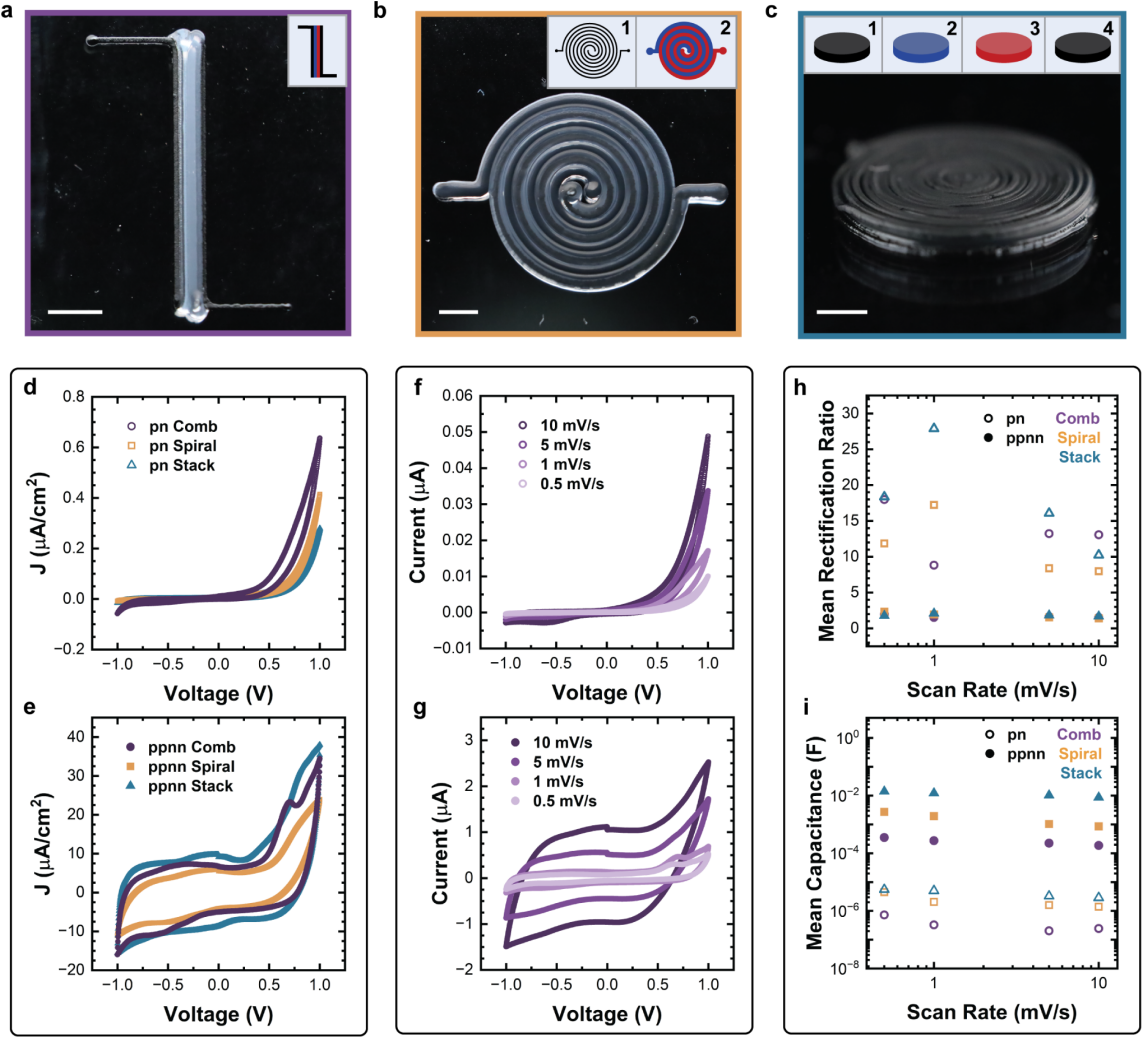
Electrical Characterization of pIL SIDs. Photographs and layer-by-layer schematics (see insets) of printed (a) comb, (b) spiral, and (c) stack SIDs. The numbers in the insets indicate the layer in which features are printed. Scale bars are 2 mm. (d) Current density (*J)* plots performed at a scan rate of 1 mV/s for representative comb, spiral, and stack SIDs printed without soft electrodes, denoted pn, and (e) with soft electrodes, denoted ppnn. Cyclic voltammetry plots for four unique (f) pn and (g) ppnn comb SIDs performed at various scan rates. Plots of (h) mean rectification ratio and (i) capacitance as a function of scan rate for all pIL SIDs (*n* ≥ 3). Error bars are not shown to improve visual clarity; the full data set is provided in Figure S6.

A classic method for understanding the performance of a diode is through cyclic voltammetry (CV).^59^ As shown in Figure 3d, CV experiments of representative pn comb, spiral, and stack SIDs measured at a scan rate of 1 mV/s reveal an asymmetric current density response that is similar to the *I–V* characteristics of traditional diodes. At negative and slightly positive potentials, the SID is “off”; current density approaches 0 μA/cm^2^ because the SID is under reverse bias, and the capacitive IDL prevents current flow. At potentials above the device-dependent threshold voltage, *V*_OCP_, of ≈0.4 V, the SID is “on”, and the current density, *J*, rapidly increases as the SID enters forward bias and transiently allows current flow. Scanning the voltage below the threshold voltage restores the IDL and reduces the current density toward zero. The rectification ratio, a figure of merit useful for comparing diode performance, is the ratio of the maximum, *I*_on_, and minimum, *I*_off_, currents achieved in a single CV curve. Importantly, the representative pn comb, spiral, and stack SIDs shown in Figure 3d exhibit strong rectification responses with rectification ratios of 11.0, 17.8, and 46.5, respectively. These values are comparable to rectification ratios of assembled SIDs reported in the literature (see Table S4). The high variability of rectification ratio between pn SIDs is primarily driven by *I*_off_. Even small changes in *I*_off_ can dramatically change the rectification ratio. However, the similarity in the shape of the asymmetric *J*–*V* response and *I*_on_ between pn SIDs of different geometries suggests that *J* is primarily dependent on the quality of the printed heterojunction rather than the interface architecture.

The *J–V* curves for ppnn SIDs (Figure 3e) show a similar threshold *V*_OCP_ of ≈0.4 V to pn SIDs but with a ≈60x increase in current density. Compared to pn SIDs, a strong hysteresis emerges in the *J–V* responses for ppnn SIDs with the inclusion of printed soft electrodes. Electrochemical impedance spectroscopy (EIS) experiments for pn and ppnn comb SIDs highlight the significant increase in capacitance when SIDs are printed with electrode inks (C_eff_ ≈3600 μF/cm^2^) compared to without the electrode inks (C_eff_ ≈2 μF/cm^2^) (Figure S7, Table S1). Therefore, the hysteresis in Figure 3e arises due to the high capacitance in ppnn SIDs. Higher capacitance systems require more time to reach a steady electrochemical state, which exceeds the time allotted with the current scan rate of 1 mV/s. *Q–V* analysis reinforces the high capacitance of the electrode ink. To see an asymmetric response, ppnn stack SIDs require >15x the amount of time to charge compared to pn stack SIDs (Figure S8). As a consequence, *I*_off_ remains large and limits the rectification ratio for ppnn comb, spiral, and stack SIDs to 2.2, 2.2, and 2.4, respectively. Again, since the formation of the heterojunction interface is identical for pn and ppnn SIDs, the decrease in the rectification ratio can be attributed to the electrochemical characteristics of the electrode inks.

We probed the scan rate-dependent performance of rectification directly by measuring *I–V* curves for pn comb (Figure 3f) and ppnn comb SIDs (Figure 3g) at scan rates of 10, 5, 1, and 0.5 mV/s. As the scan rate decreases, *I*_on_ and *I*_off_ decrease by ≈7x and ≈5x, respectively. This trend is reflected in Figure 3g for ppnn SIDs as well, where *I*_on_ and *I*_off_ decrease by ≈6x and ≈5x, respectively. Notably, the hysteresis significantly decreases as the scan rate decreases for ppnn comb SIDs. Slower scan rates allow more time for ions to move through the SID but can also lead to saturation in non-Faradaic devices, which lowers the net current.^60^ Similar scan rate-dependent trends also appear in spiral and stack SIDs (Figure S9).

To better visualize the impact of scan rate on rectification performance, Figure 3h shows the mean rectification ratio (*n* ≥ 3) of each SID architecture as a function of scan rate from 10 mV/s to 0.5 mV/s. For pn SIDs at faster scan rates of 10 and 5 mV/s, the mean rectification ratio is ≈8–13 for each geometry. As shown in Table S2, the rectification ratio for pn SIDs varies between printed devices, so it is difficult to extract precise insights on any geometrical factors that influence rectification at high scan rates. Slower scan rates magnify differences in time-dependent rectification that arise from print variability and/or geometrical factors between different architectures. For pn comb SIDs, the mean rectification ratio decreases from 13.2 ± 4.7 to 8.8 ± 2.7 and then increases to 18.0 ± 5.6 as the scan rate decreases from 5 to 0.5 mV/s. However, for pn spiral and stack SIDs, the highest mean rectification ratio occurs at 1 mV/s (17.2 ± 13.0 and 27.9 ± 16.4) and then decreases for the 0.5 mV/s experiments (11.8 ± 6.5 and 18.3 ± 7.0). For all ppnn SIDs, the mean rectification ratio shows a slight increase for slower scan rates but remains close to a mean rectification ratio of ≈2 across all evaluated scan rates (see data available in Table S2). Even at low scan rates, the rectification ratio of ppnn SIDs remains low. Future work is needed to decouple the time-dependent response of SIDs from the electrode material.

One possible source of variability in our process can arise from the SID alignment. The rectification ratio is highly susceptible to even small deviations of the location of the heterojunction interface with respect to the planar ITO electrodes. As shown in Figure S10, shifting the centerline of the heterojunction toward the p-type or n-type side artificially strengthens the applied electric field on one side of the SID and results in large changes to the rectification ratio. While there may be an optimal scan rate for SIDs, which we suspect is around 1 mV/s for ours, the variability in the rectification ratio makes it difficult to identify its precise value. Further experiments that investigate the scan-rate-dependent rectification ratio of SIDs with distinctly different mobile ions may be needed, as we suspect that optimal scan rates are related to the mobility of the free ions.

Figure 3i provides the mean capacitance as a function of scan rate for the same SIDs presented in Figure 3h. For pn SIDs, the mean capacitance values range from 0.2 ± 0.1 μF to 5.5 ± 0.7 μF, while those for ppnn SIDs range from 190 ± 30 μF to 14.0 ± 2.5 mF. Overall, the mean capacitances of ppnn SIDs are ≈3 orders of magnitude higher than those for pn SIDs. The capacitance slightly increases as the scan rate decreases for all SIDs. However, the amount of material present in the SID, particularly the electrode material, has a dominant impact on the mean capacitance. We see this effect when comparing the stack and comb SIDs: the mean capacitance is highest for stack SIDs and lowest for comb SIDs, which contain the largest and smallest volumes of electrode ink, respectively.

With chronoamperometry (CA), we investigated the time-dependent performance of SIDs more quantitatively than is possible with CV (Figure 4). Figure 4a illustrates the components of a typical CA experiment, where rectification is characterized over time under an AC potential. First, we equilibrate the device at the experimentally determined average *V*_OCP_ of −0.35 V for 10 min to establish a consistent baseline for all SIDs. The equilibration period is introduced to reduce device-to-device variability that arises from printing. After equilibrating, we measure SID rectification under a square wave AC voltage for 2 h with amplitude *V*_pk_ = ±1 V and frequency *f* = 0.01 Hz. Each cycle has both a positive and negative half cycle and repeats every 1/*f* s for the duration of the 2-h period. Similar to CV, the rectification ratio in CA is the ratio of the minimum current from the positive half cycle, *I*_on_, and the maximum current from the negative half cycle, *I*_off_. To probe the dynamic, history-dependent response of SIDs, we introduce a 30-min reset period into the CA experiment, in which a strong reverse bias is held at −*V*_pk_ across the SID in an attempt to restore the mobile ions to their initial positions prior to the start of the measurement. Finally, we reapply the AC potential to measure the performance difference after the reset period.

**Figure 4:**
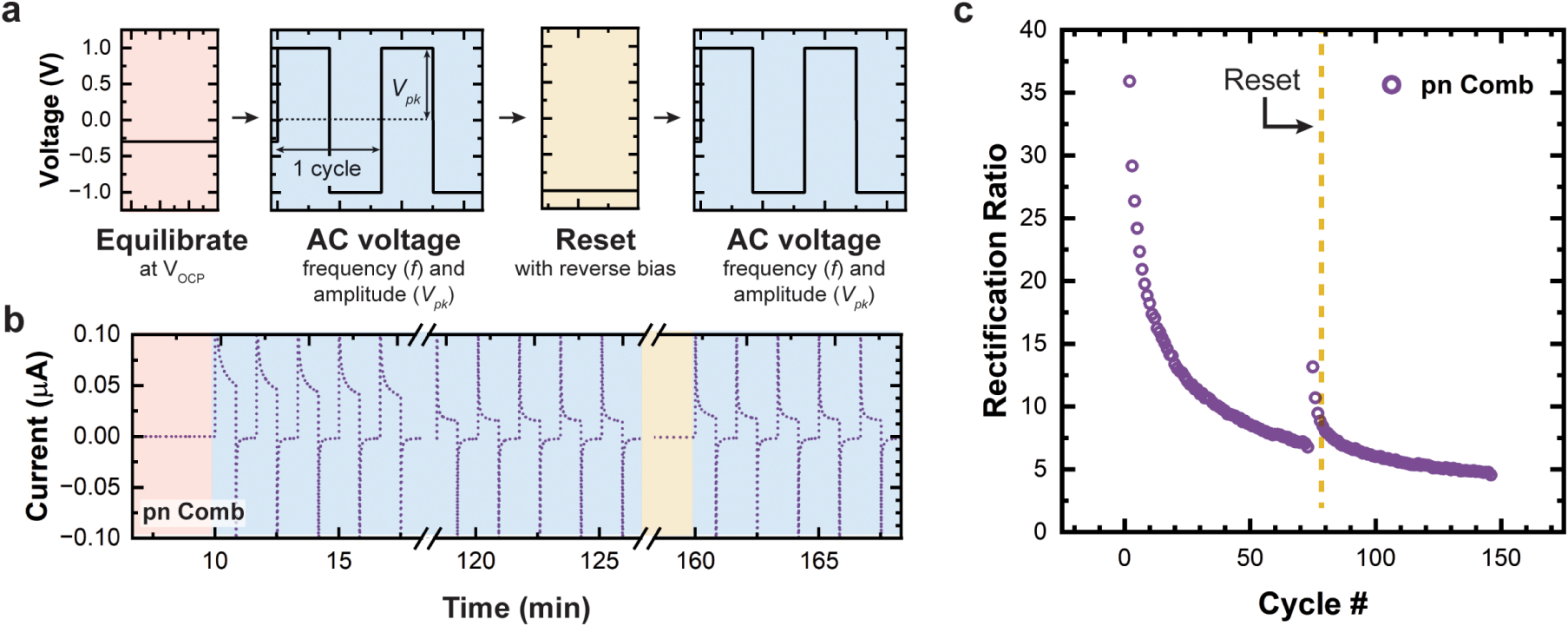
pIL SID Rectification. (a) A chronoamperometry (CA) experiment for a SID involves four steps: equilibrate the SID at the open-circuit potential (*V*_OCP_, red), apply an AC potential alternating between +1 V and −1 V at a fixed frequency (blue), reset the SID by holding it under reverse bias (yellow), and repeat the application of an identical AC potential (blue). (b) As CA voltages are applied to the pn comb SID (at 0.01 Hz), we record their current (*I*) response over time (*t*). The corresponding rectification ratios of the pn comb SID at each cycle of the CA experiments are shown in (c), where the reset period is indicated in cycle count by the dashed yellow line.

Combining all of the blocks of the experiment together results in the *I-t* response curve shown in Figure 4b. Only the first and last five cycles of the AC voltage period are displayed for clarity. In the first five cycles of Figure 4b, the pn comb exhibits a strong rectification response. For each cycle, the current is positive when the applied voltage is +1 V, and the current quickly approaches 0 μA when the voltage is −1 V. As the experiment continues in the first AC voltage segment, *I*_on_ decreases from 0.051 μA to 0.015 μA between the first and last (i.e., 72nd) cycles, while *I*_off_ has only slightly decreased from −0.0014 μA to −0.0022 μA. The drop in *I*_on_ drives a similar trend in the rectification ratio for the first 72 cycles before the reset segment (Figure 4c). The reset temporarily increases *I*_on_ and decreases *I*_off_, but only for ≈15 cycles before the currents return to their values prior to the reset. Overall, we evaluated our SID performance over 200 min of operation under this AC voltage input. Performing an identical CA experiment on ppnn combs shows a similar performance over time, but with lower values for the rectification ratio (Figure S11). Consistent with Figures 4 and S11, CA experiments for spiral and stack geometries differ in their rectification values but still show decreases in their rectification ratios (Figure S12).

Figure 4c reveals that the SID rectification ratio decreases over time, and strong reverse bias potentials are insufficient for fully restoring the initial rectification ratio. As shown in Figure S13, a loss in the SID rectification ratio also manifests in CV experiments. Repeated CV measurements of comb SIDs at room temperature lead to an *I*_on_ that degrades up to 30% of the initial value after 1 week (Figure S13a,b). At 80^◦^C, the curing temperature for traditional thermally activated initiators, a near-complete loss of rectification occurs after only 24 h (Figure S13c,d).

Overall, our characterization of SID performance is more extensive than what is typically reported in the literature. As indicated in Table S4, the stability of SID rectification is rarely reported for longer than a few minutes. Consequently, this change in rectification performance is not well understood and hinders the integration of SIDs into long-term iontronic applications. Incomplete polymerization may be one contributor. In traditional assembly-based manufacturing, oxygen inhibition is minimized by curing via capillary infill between glass slides, and residual monomers can be removed by washing with solvent.^61^ However, our ambient printing process exposes extruded inks to oxygen, and solvent washing can swell the device, particularly for complex 3D geometries. A CV experiment on planar comb SIDs before and after washing with dichloromethane suggests that removing residual monomer does not improve rectification (see Figure S14). Furthermore, ATR-FTIR experiments reveal that monomers are not fully polymerized in our printed inks (Figure S15). As a result, we expect slight mixing of unreacted monomer to occur at the pIL heterojunction, which may destabilize the ability of an IDL to form over time. Future investigations that quantitatively measure the capacitance change of the IDL over time via EIS can shed light on this phenomenon but will require electrode modification to resolve the IDL from the EDL peak (see Figure S16 and Table S3). Strategies for mitigating oxygen inhibition and maximizing monomer conversion may be necessary for stabilizing rectification in 3D printed SIDs.

Finally, the decrease in the rectification ratio for ppnn SIDs can be attributed to the electrochemical characteristics of the electrode inks. It is challenging to develop an extrudable electrode ink that has minimal impact on the rectification ratio of ionic diodes. Metals such as copper introduce nondesirable Faradaic reactions (Figure S17). Microporous carbon adhesives commonly used for assembly-based methods are not compatible with extrusion.^25,37^ While the low rectification ratio in ppnn SIDs is not suitable for fast, responsive diode applications like computation, the ≈60x increase in current density and high capacitance imparted by the electrode ink can be beneficial for applications in ionic energy storage,^11^ where the existence of a heterojunction helps maintain stored charge until release is needed. Further material development is needed to design electrode inks that can enable the measurement of high rectification ratios in fully soft ionic diodes.

### Fully Printed Multi-SID Devices

Advanced iontronic devices for bioinspired sensing, neuromorphics, and soft robotics will require devices with arrays of SIDs in complex 3D architectures that are impractical, if not impossible, to fabricate with existing manufacturing methods.^9,19,62,63^ Moreover, multi-SID devices will require more consistent performance and methods for integrating individual SIDs than manual assembly processes can produce. A key motivation for additively manufacturing SIDs is to reduce the time required for multi-SID device fabrication without sacrificing geometric complexity. We used our materials and 3D printing method to fabricate multi-SID devices that are difficult to achieve otherwise: multi-comb SID devices with tunable current responses and open-circuit potentials, and dense SID arrays with up to 900 ppnn stack SIDs at 100 SIDs/cm^2^ (Figure 5).

**Figure 5:**
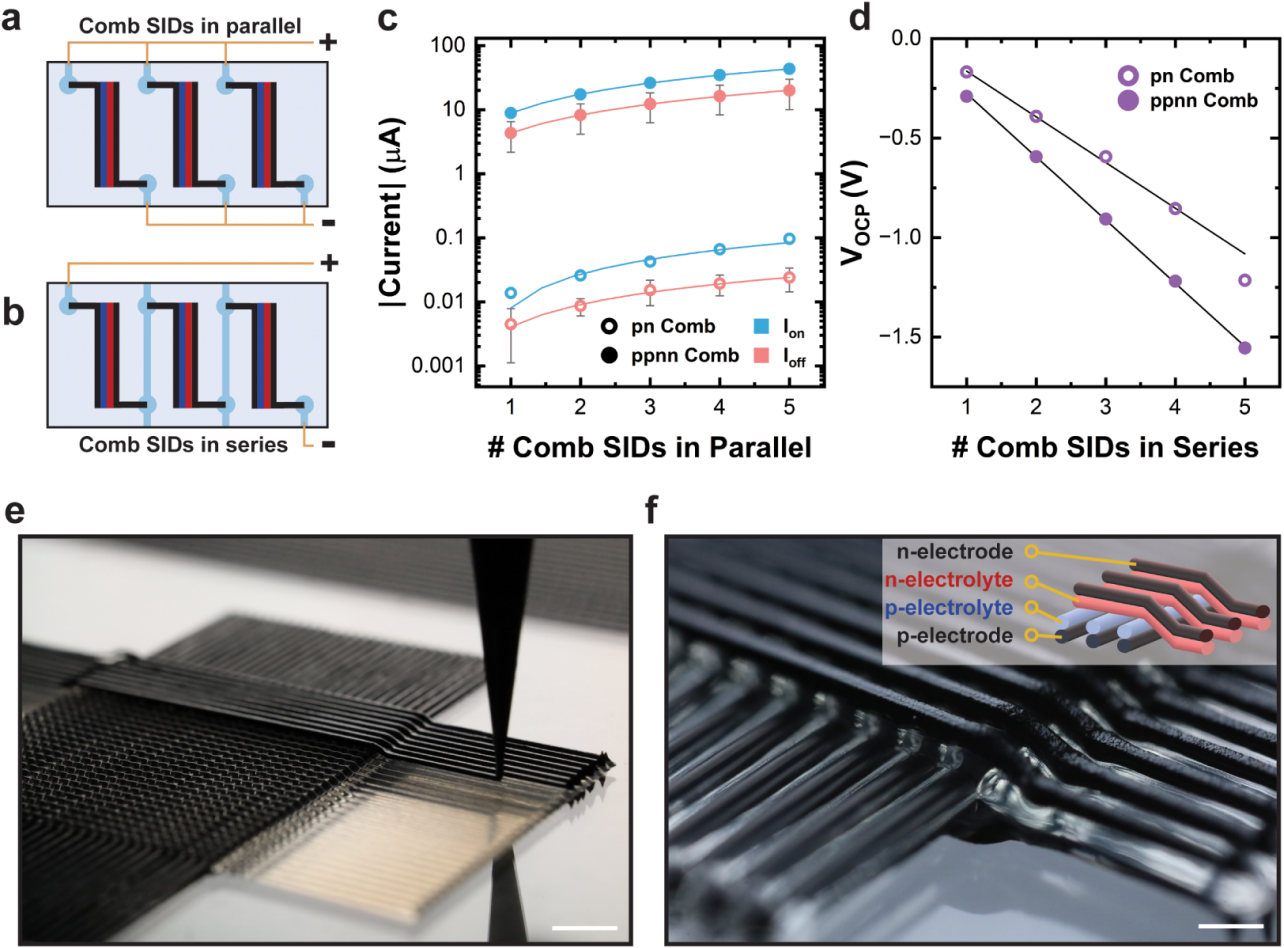
Printing Multi-SID Circuits. Schematics of 3-comb SID devices connected in (a) parallel and (b) series configurations. (c) Plot of *I*_on_ and *I*_off_ versus the number of comb SIDs in parallel. The current values are taken from CA data at *V*_pk_ = ±1 V and *f* = 0.1 Hz, averaged across the first 5 cycles. Error bars for *I*_on_ are smaller than the plotted points. (d) Plot of open-circuit voltage (*V*_OCP_) versus the number of comb SIDs in series. *V*_OCP_ is averaged across 10 s, and the error is smaller than the plotted points. The trend lines shown in (c) and (d) are linear fits of the corresponding data. (e) Photograph of a 20 × 20 mm^2^ SID array midprint. Scale bar is 5 mm. (f) Inset of a fully printed SID array with a schematic representation of the printed array structure. Scale bar is 1 mm.

A straightforward iontronic device architecture is one where multiple SIDs are configured together in parallel (Figure 5a) or series configurations (Figure 5b) for tailored current and voltage responses.^64^ Similar to parallel configurations of multiple batteries, we observe that SIDs printed in parallel configurations each experience a similar voltage drop when a potential is applied across the shared external circuit. Figure 5c shows *I*_on_ and *I*_off_ from CA experiments (*V*_pk_ = ±1 V and *f* = 0.1 Hz) for pn and ppnn comb SIDs connected in parallel. The plotted *I*_on_ and *I*_off_ values are averaged across the first five cycles of each CA experiment. Current responses were ≈3 orders of magnitude larger for devices with ppnn comb SIDs compared to pn combs. For pn comb arrays, *I*_on_ increased by a factor of 6.9x from 0.014 μA to 0.096 μA as SIDs were added in parallel from one to five. Similarly, *I*_on_ for ppnn comb arrays increased 4.9x from 8.85 μA to 43.3 μA. The current scales linearly for pn and ppnn comb SIDs in parallel, with a slope of 0.019 and 8.60, respectively. This scaling follows Kirchhoff’s current law, where *I*_TOT_ = n*I_SID_, and *I*_SID_ is the current through one SID and *n* is the number of SIDs in parallel. As anticipated, there is a negative correlation between comb SIDs connected in series and current (Figure S18a). Conversely, when comb SIDs are printed in series, the voltage drop across each device, *V*_OCP_, is additive according to Kirchhoff’s laws. The *V*_OCP_ arises from the chemical potential gradient across the heterojunction in the SID.^16^ Figure 5d shows that *V*_OCP_ decreases linearly with a slope of −0.23 for pn combs and −0.32 for ppnn combs as the number of comb SIDs in series increases from one to five. In-series ppnn comb devices exhibit *V*_OCP_ of higher magnitude than for pn combs. In contrast, comb SIDs connected in parallel retain a constant *V*_OCP_ (Figure S18b).

An alternative way to view these scaling relationships is through CV, as shown in Figure S19. Combs connected in parallel increase the net current in forward bias but retain the same electrochemical window. Instead, connecting combs in series increases the *V*_OCP_ and broadens the electrochemical window. The trends shown in Figure S19 are consistent with those in Figure 5. However, CV also captures an increase in capacitance as SIDs are connected in parallel, as indicated by the wider hysteresis for additional combs in parallel. Printing in series and in parallel SID assemblies, as we demonstrate, is one rapid way of tailoring *I–V* characteristics for specific iontronic circuit needs without changing material properties.

Since our inks exhibit a stiffness and yield-stress nature that supports multi-layer printing, we 3D printed crossbar-inspired arrays^65^ of diodes, as shown in (Figures 5e,f). We can print these device architectures following similar steps used to print 3D stack SIDs. First, the p-type electrode and electrolyte inks are sequentially printed as multiple, vertically stacked traces. After photocrosslinking the p-type electrolyte, the n-type electrolyte is printed orthogonally to the p-type ink traces, which serve as bridging supports (Figure 5e). The n-type electrolyte is photocrosslinked, and the n-type electrode inks are printed on top of the n-type electrolyte traces. The entire device is thermally cured to crosslink the electrodes. The fully cured array forms individual SIDs at each point of intersection of the p-type and n-type components, which have an approximate heterojunction interface area of 200 × 200 μm^2^ (Figure 5f). Each diode in the multi-SID array is individually addressable; as shown in Figure S20 for a smaller, silicone-encapsulated device, we measured a rectification ratio between 1.5 and 4.5 for the 10 SIDs along the main diagonal along the array. We note that SID-to-SID variability of the rectification ratio in this demonstration could also come from the placement of manually inserted electrodes. Overall, this device architecture demonstrates the capabilities of our method to fabricate complex, densely populated SID-based assemblies that have potential use for integrated iontronic systems, like spatially resolved, self-powered sensing skins.^16,19^

## Conclusion

We have presented a material design and multimaterial manufacturing strategy for fully printing SIDs from polymerizable ionic liquids. We demonstrate several examples of complex SID geometries that can be printed with all of the required p- and n-type polyelectrolyte and electrode components. The geometry-, scan-rate-, and time-dependent rectifying capabilities of our SIDs were characterized using several electrochemical techniques. Altogether, our printed diodes exhibit maximum rectification ratios of 46 and 3 when printed without and with our electrode inks, respectively, which is on par with previously reported ionic diodes (Table S4). We also 3D printed multi-SID devices that have not been previously reported, highlighting the opportunities additive manufacturing creates for tuning current and voltage responses and fabricating dense arrays of miniaturized SIDs for future iontronic applications.

Importantly, our pIL SIDs do not contain solvents or rely on hydrolysis to function, as many hydrogel-based SIDs do. We circumvent water loss, which leads to hydrogel-based device degradation and variations in performance over time (Figure S1). We also note that our SIDs rectify at rates similar to those of other hydrogel- and pIL-based SIDs. However, achieving stable and high rectification remains a challenge to be addressed in future work. To achieve higher rectification ratios, improvements in the electrode inks that enable rapid ion rearrangement under applied potentials without imparting additional EDL capacitance are needed. Moreover, chemical strategies that enable complete polymerization and cross-linking, even under ambient conditions, are needed to stabilize rectification. We anticipate that the highly tunable chemical and mechanical properties of pILs^48,49^ will accelerate future improvements to the printed SID performance and capabilities. Altogether, this work lays a foundation for rethinking how iontronic devices are additively manufactured for applications such as bioinspired sensing,^1,4^ computation,^9,38^ signal processing,^5,24^ and energy production.^11^

## Experimental Section

### Materials

Unless otherwise stated, all purchased materials were used without further purification. All solvents, other than nanopure water, were purchased from Sigma-Aldrich.

#### Synthesis of 1-Ethyl-3-methylimidazolium (3-Sulfopropyl)­acrylate

ES was synthesized according to previous protocols.^53,66^ In a 3-L round-bottom flask, solid 1-ethyl-3-methylimidazolium chloride (500.0 g, 3.41 mol, [EMIM]­Cl, Ambeed) and a catalytic amount of 2,6-bis­(1,1-dimethylethyl)-4-methylphenol (0.75 g, 0.003 mol, BHT, Sigma-Aldrich) were mixed in 1 L of acetonitrile and stirred until dissolved with an overhead stirrer (Velp Scientific). To the stirring [EMIM]Cl solution, (3-sulfopropyl)­acrylate potassium salt (792.2 g, 3.41 mol, K­[SPA], Sigma-Aldrich) was added slowly. The pale yellow reaction mixture was vigorously stirred overnight at room temperature. The fine white precipitate (potassium chloride, KCl) was filtered via Buchner filtration over a medium-grain glass frit to yield a pale yellow solution. Acetonitrile was removed via rotary evaporation at 45^◦^C. The residual viscous yellow liquid was redissolved in an excess of DCM (>1 eq by volume) and allowed to chill overnight in a 5^◦^C fridge. The solution was filtered again to remove a very fine yellow precipitate, yielding a pale yellow liquid. The product was stirred for 1 h with MgSO_4_ and activated charcoal (decolorizing agent). A final quick filtration followed by complete removal of DCM yielded a viscous, faintly yellow liquid. Purity was confirmed by ^1^H NMR (Figure S21).

#### Synthesis of 1-[2-Acryloyloxyethyl]-3-butylimidazolium Bis­(trifluoromethane)­sulfonimide

AT was synthesized according to previous protocols.^53,67^ In 750 mL of acetonitrile, 2-bromoethyl acrylate (300.0 g, 1.68 mol, Ambeed) was added along with 1-butylimidazole (212.3 g, 1.71 mol, TCI Chemicals) and stirred under mild reflux (60^◦^C) overnight in argon. Acetonitrile was removed using rotary evaporation, and the product, 1-[2-acryloyloxyethyl]-3-butylimidazolium bromide ([AEBI]­Br), was extracted with nanopure water and washed at least three times with dichloromethane (DCM). The aqueous phase was collected, and a catalytic amount of 2,6-bis­(1,1-dimethylethyl)-4-methylphenol (0.3716 g, 0.002 mol, BHT, Sigma-Aldrich) was added and magnetically stirred. Separately, bis­(trifluoromethane)­sulfonimide lithium (481.1 g, 1.68 mol, Li­[TFSI], Ambeed) was dissolved in nanopure water and subsequently added to the [AEBI]Br solution while stirring. Upon addition of Li­[TFSI], the product immediately formed an organic phase. The mixture was stirred at room temperature for several hours. AT was extracted with DCM and washed with nanopure water until the washing liquid passed a 0.5 M AgNO_3_ test. The faintly yellow product was dried over MgSO_4_ and decolorized with activated charcoal. MgSO_4_ and activated charcoal were filtered out, and the remaining DCM was evaporated to yield AT as a slightly viscous, colorless liquid (676.2 g, yield 80%). Purity was confirmed by ^1^H NMR (Figure S22) and ^19^F-NMR (Figure S23).

#### Preparation of p-/n-Type Electrode Inks

ES/AT (p-type/n-type) IL monomers were mixed with 0.4 mol % PEGDA (*M*_n_ 250, Sigma-Aldrich) and 14 wt % acetylene black nanoparticles (50% compressed, Alfa-Aesar) in a Flacktek Speedmixer (DAC 330) for five cycles at 3500 rpm for 150 s. Electrode inks were chilled in a 5^◦^C fridge for 5 min between mixing cycles. After 5 mixing cycles, 0.4 mol % thermal initiator V-70 (Fuji-Film Wako Chemicals) was added to the ink. The ink was mixed for another two cycles at 3500 rpm for 150 s; the ink was chilled for 10 min between mixing cycles and after the last cycle. The final inks were smooth, thick black pastes with no visible clumps. Inks were loaded into 3 cm^3^ syringe barrels (Nordson EFD) by centrifugation (Megafuge 8R, Thermo Fisher). Commercially available tapered nozzles (SmoothFlow 200 μm inner diameter, Nordson EFD) were fixed to syringe barrels before printing.

#### Preparation of p-/n-Type Electrolyte Inks

The p-type electrolyte ink was made by combining the IL monomer, ES, with 0.5 wt % PEGDA, 0.5 wt % TPO-L photoinitiator (Breckenridge Technologies), and 9 wt % Aerosil R 106 fumed silica (Evonik) in a container. Similarly, the n-type electrolyte ink was made by combining the IL monomer, AT, with 0.5 wt % PEGDA (Sigma-Aldrich, *M*_n_ = 250), 0.5 wt % TPO-L photoinitiator (Breckenridge Technologies), and 6 wt % Aerosil E 805 fumed silica (Evonik) in a container. The inks were mixed in a Flacktek Speedmixer for two cycles at 3000 rpm for 210 s. The final inks were a clear gel. The inks were loaded into 3 cm^3^ syringe barrels (Nordson EFD) by centrifugation (Megafuge 8R, Thermo Fisher). Commercially available tapered nozzles (SmoothFlow, 200 μm inner diameter, Nordson EFD) were fixed to the syringe barrels before printing.

#### Preparation of p-/n-Type Hydrogels

Hydrogel formulations were modified from previous protocols.^20^ The p-type hydrogel was made by combining (3-sulfopropyl)­acrylate potassium salt (2.210 g, Ambeed), acrylamide (2.5 g, Sigma-Aldrich), N,N’-methylenebis­(acrylamide) (30 mg, Sigma-Aldrich), lithium phenyl-2,4,6-trimethylbenzoylphosphinate (25 mg, Sigma-Aldrich), and 6.83 mL of Milli-Q water. The n-type hydrogel was made by combining 2-acryloxyethyltrimethylammonium chloride (3.050 g, 80 wt % in H_2_O, Ambeed), acrylamide (2.5 g, Sigma-Aldrich), N,N’-methylenebis­(acrylamide) (30 mg, Sigma-Aldrich), lithium phenyl-2,4,6-trimethylbenzoylphosphinate (25 mg, Sigma-Aldrich), and 6.22 mL of Milli-Q water. Hydrogels were formed through capillary infill between glass slides coated with PTFE tape with ≈400 μm PDMS spacers. Hydrogel solutions were photocured (EnvisionTEC PCA 2000 Light Curing Box) for 1 h. After polymerization, hydrogels were cut into ≈7 mm squares and manually stacked between ITO for electrical measurements.

#### Multimaterial DIW 3D Printing

A custom, high-precision gantry designed for multimaterial 3D printing (AGS15000 gantry, Aerotech Inc., Pittsburgh, PA, USA) was used for all DIW printing. The gantry is equipped with four independently controllable z-stages to which the inks were mounted. Print paths were hand-designed using open-source Python packages (mecode.py^68^ modified to incorporate better handling of multimaterial designs). Python scripts written in-house using mecode generate a combination of G-code and Aerobasic commands that coordinate the motion of the gantry with external equipment.

Prior to printing, nozzles were aligned with a pair of optical micrometers (Keyence LS-7010) mounted 90^◦^ from each other on the gantry stage. Custom Aerobasic scripts were written to automate the alignment process by measuring each nozzle, writing X, Y, and Z alignment values to a data file, and reading the alignments during printing. To measure a nozzle attached to a Z-stage, the nozzle is translated to a predefined standard position above the center of the micrometer readheads. The Z-stage lowers in 1 mm increments until the nozzle passes through the measurement plane of the first micrometer. Next, the Z-stage raises 1 mm upward and then resumes translation downward in 1 μm increments. Upon passing through the measurement plane again, the center of the nozzle (in the *X*-direction) is adjusted to the center of the readhead within ±1 μm. After correcting the X-alignment, the Z-stage continues translating down in 1 mm increments until reaching the second readhead measurement plane. Similarly, the Z-stage raises 1 mm, moves down at a step size of 1 μm, and then aligns the center of the readhead and the nozzle to correct the Y-alignment. The current X, Y, and Z position is read from the Aerotech controller and written to a data file. This alignment process is repeated for all four nozzles and serves as an absolute reference position for automatic nozzle switching. The first Z-stage material was aligned to the substrate by hand to find the relative start point for printing.

Inks were extruded sequentially using digital pressure regulators (Ultimus V, Nordson EFD) onto an ITO glass substrate (MSE Supplies, 40–60 Ω/sq) at pressures ranging from 50 to 70 psi for electrode inks and 15–35 psi for electrolyte inks, depending on the rheological behavior of the ink batches (Video S1). Electrolyte inks were cured for 15 s at 1 W/cm^2^ midprocess with a UV spot curing system (365 nm, Omnicure, Excelitas). Curing time was validated through photorheology (Figure S24). After printing, diodes with thermally curable electrodes were cured at 80^◦^C in an argon-filled glovebox (MBraun) for 5 min. For Figure S14 only, fully cured, printed comb SIDs were rinsed with dichloromethane and dried at 60^◦^C for 30 min.

#### Rheological Characterization

p-/n-type electrode and electrolyte inks, and pure IL monomers, were rheologically characterized by using a strain-controlled rheometer (MCR 302e, Anton Paar) with a 25 mm parallel plate measuring head at a 200 μm gap. Flow curves were measured using a logarithmic shear rate sweep from 0.001 s^–1^ to 100 s^–1^. Storage (G’) and loss (G″) moduli were measured using an oscillatory amplitude sweep at a constant frequency of 1 Hz and logarithmically increasing shear strain from 0.001% to 100%. The yield stress was defined as the crossover point between G’ and G″. Photorheology experiments were measured with a constant shear strain of 0.1% with a constant frequency of 10 Hz (Figure S24). After 20 s, the material was exposed to 1 W/cm^2^ of UV light (365 nm, Omnicure, Excelitas) through a quartz plate from below the rheometer stage until G’ reached a plateau.

#### Electrochemical Characterization

All electrochemical characterization tests were performed within 24 h after printing. Unless stated otherwise, the positive (i.e., working) electrode was connected to the p-type side of each device, and the negative (i.e., counter and reference) electrode was connected to the n-type side. Cyclic voltammetry (CV) curves were measured using a PalmSens4 with a step size of 0.00244 mV/step, and sweeping in the positive direction from 0 V to +1 V for at least two scans. The first CV scan was disregarded. Chronoamperometry experiments were measured with a Keithley 2400 Sourcemeter, using a custom-written Python script to externally control and program the instrument. Electrochemical impedance spectroscopy (EIS) experiments were performed at *V*_OCP_ on a PalmSens4 instrument with a frequency range of 1 MHz to 0.01 Hz and an amplitude of 50 mV. For EIS measurements performed at applied DC biases, the DC bias was applied for 5 min prior to measuring EIS. EIS measurements were fitted with the PSTrace software provided by PalmSens using the Levenberg–Marquardt fitting algorithm. All fitting was done using unconstrained elements with equivalent circuits described in the literature.^16,24^

#### Multi-SID Characterization

All multi-SID characterization experiments were performed on devices printed on the same day. For pn combs in series/parallel and ppnn combs in parallel, pogo pin probe clips (Adafruit Industries) were attached to ITO contacts on the prepatterned glass. Jumper cables from the pogo pin probe clips were used to join the devices together through a breadboard. The ppnn combs were connected in series directly via the prepatterned ITO glass. Optically photographed SID crossbar-inspired arrays were printed on normal glass substrates. Electrically measured SID arrays were printed on silicone substrates and encased with additional photocurable silicone to enable the integration of stable electrical contacts. The photocurable silicone was prepared by mixing 15 g of low molecular weight vinyl-terminated silicone (DMS V21, Mn = 6000, Gelest Inc.), 22.5 g of high molecular weight vinyl-terminated silicone (DMS V52, Mn = 15500, Gelest Inc.), 3.086 g of hexafunctional thiol crosslinker (SMS-142, Gelest Inc.), 18.516 g of difunctional thiol crosslinker (SMS-022, Gelest Inc.), and 0.1314 g of photoinitiator (TPO-L, Breckenridge Technologies) until homogeneous. Silicone monomer was cast in a 3D-printed mold, sandwiched by an ethyl acetate sheet to create a flat surface, and photocured for 10 min. After printing multi-SID arrays on the PDMS substrate, an additional silicone monomer was cured on top in the same way as before. Stainless steel beading needles (0.4 mm diameter) with silver wire were used as electrical contacts by puncturing the silicone and electrode inks. All electrochemical tests for multi-SID devices were measured using a PalmSens4.

#### Four-Point Probe Resistance

Conductivity of the p-/n-type electrode and electrolyte inks was measured using a four-point probe with rounded, soft-tipped probes spaced at 1.27 mm (Ossila). Inks were cast into 2 cm × 2 cm × 450 μm sheets. Electrolyte inks were photocured (EnvisionTEC PCA 2000 Light Curing Box) for 15 s. Electrode inks were cured at 80^◦^C in an argon-filled glovebox (MBraun) for 5 min. 100 data points were collected over 10 s in triplicate for each casted film.

#### ATR-FTIR Characterization

Samples of p-/n-type electrolyte ink were deposited onto glass slides coated with adhesive PTFE tape (McMaster). For exposed samples, the ink was blade coated to ≈400 μm. Covered samples were clamped between a second glass slide coated with PTFE tape with ≈400 μm PDMS spacers. All samples were photocrosslinked under UV light (Kessil, 365 nm) for 1 min. Exposed samples were placed face down on the ATR crystal (Thermo Nicolet Nexus 870 spectrometer) to measure the absorbance at the exposed surface.

## Supplementary Material





## Notes

Ionic liquid monomer NMR spectra; additional electrochemical characterization; extended data tables; literature review table (PDF)
Video of the printing process (MP4)
