# Supplementary material for: Fully Printed Ionic Diodes from Polymerizable Ionic Liquids
Source: ACS Nano. 2026 Jul 27;20(31):21953–64. doi: 10.1021/acsnano.6c06135 (PMC13472320; doi:10.1021/acsnano.6c06135)
Supplement: Supplementary file 1 [file nn6c06135_si_001.pdf]

Supplementary Information: Fully Printed Ionic Diodes from Polymerizable Ionic Liquids

Alexander Q. Kane,† Eric Yang,† and Ryan L. Truby*,†,‡

†*Department of Materials Science and Engineering, Northwestern University, Evanston, IL 60208*

‡*Department of Mechanical Engineering, Northwestern University, Evanston, IL 60208*

E-mail: rtruby@northwestern.edu

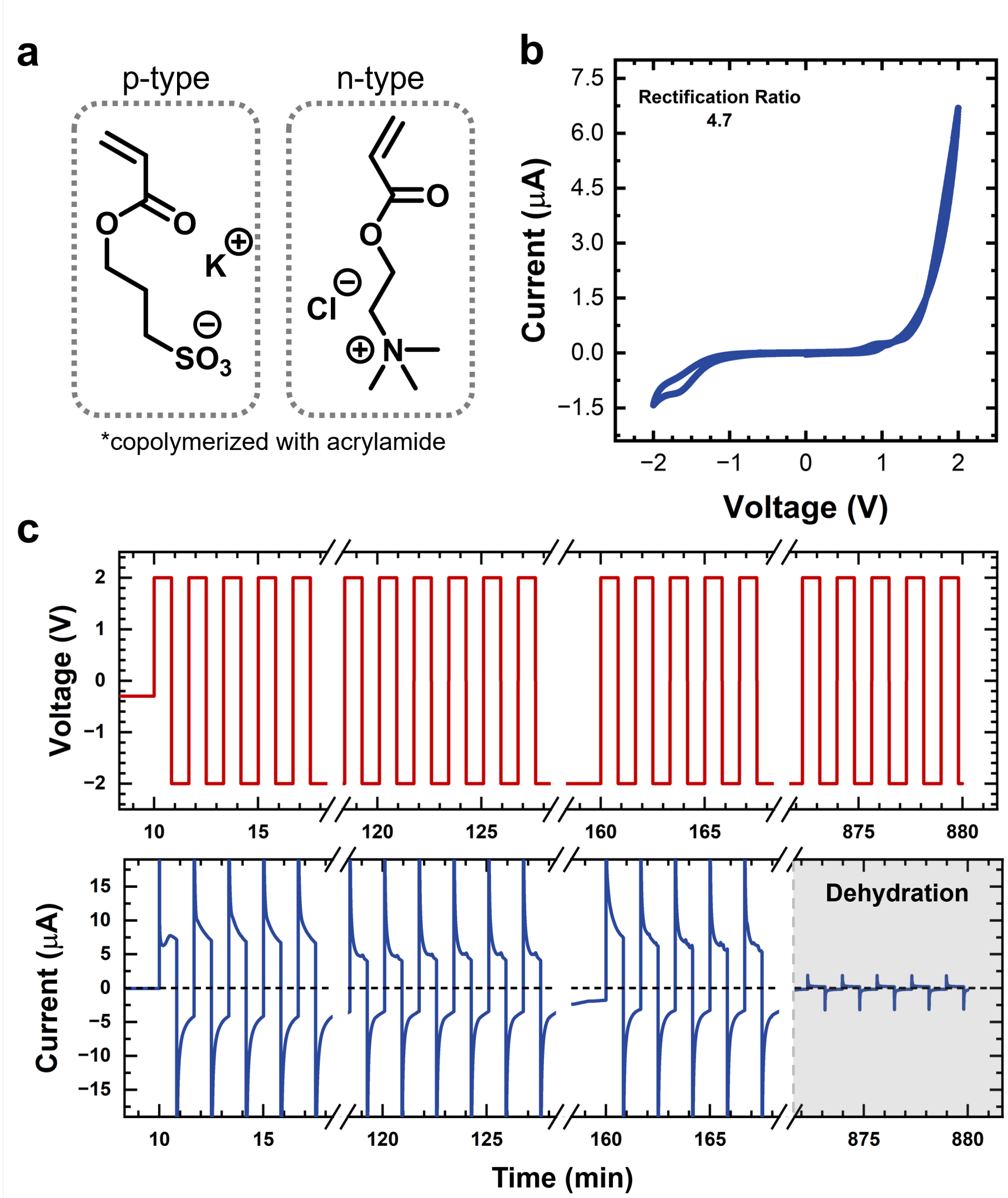

Figure S1: **Hydrogel SIDs Dehydrate Over Time.** (a) Chemical structure of p-/n-type hydrogel monomers, copolymerized with acrylamide. (b) Cyclic voltammetry of an assembled hydrogel soft ionic diode (SID) with a scan rate of 5 mV/s. (c) A chronoamperometry experiment given by the V-t curve (top) that produces an I-t response (bottom). Hydrogel SIDs lose water rapidly during the experiment, resulting in a drop in overall current.

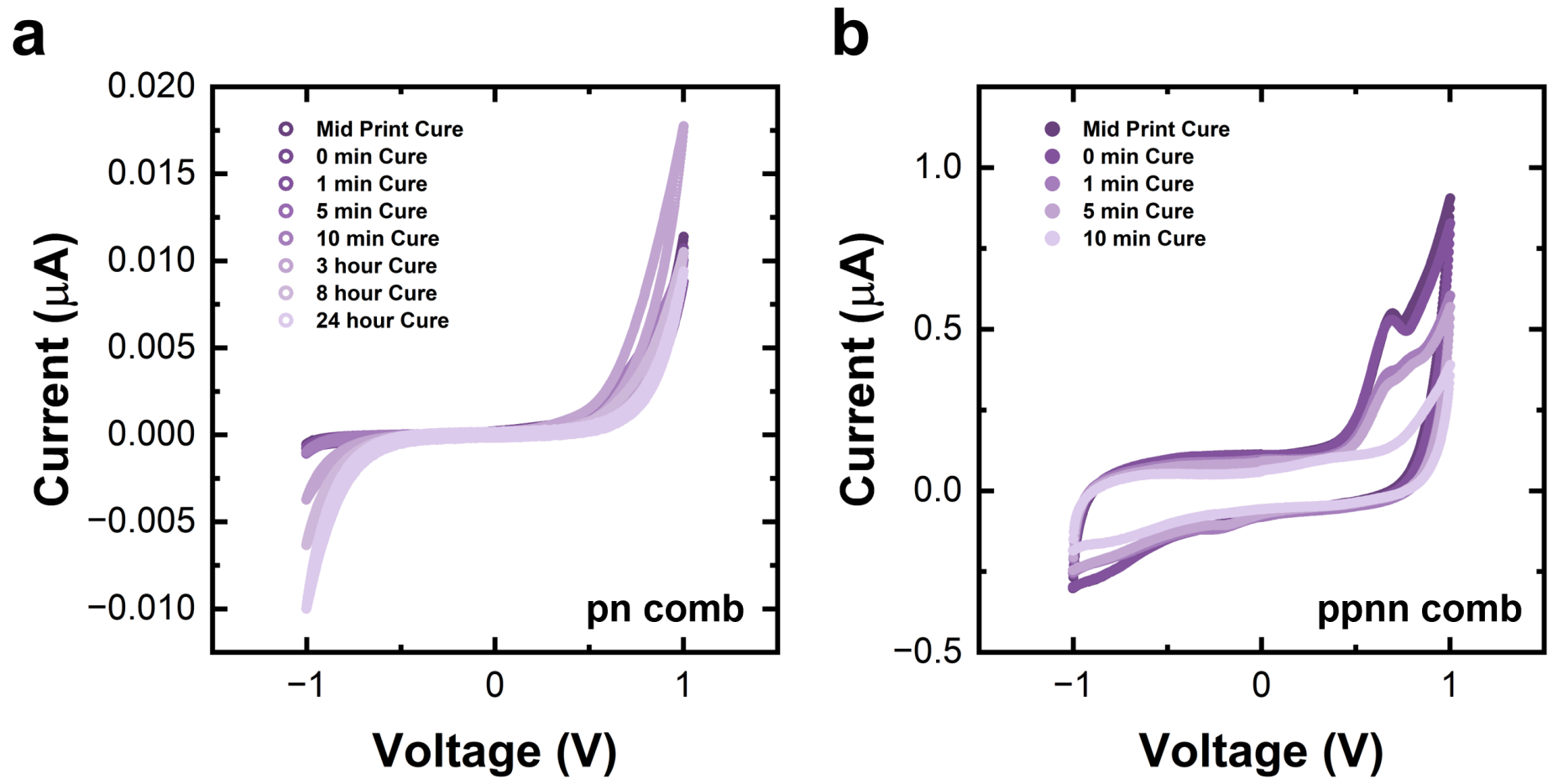

Figure S2: **UV Crosslinking Prevents IL Monomer Mixing.** Cyclic voltammetry of (a) pn and (b) ppnn comb SIDs, where UV curing is performed on both electrolyte layers simultaneously X min after print completion (i.e., an X min Cure). The p-type electrolyte is cured before printing the n-type electrolyte, as depicted in Figure 1 for the Mid Print Cure. Monomer mixing between p-/n-type electrolyte layers that occurs while waiting to cure dramatically hinders rectification.

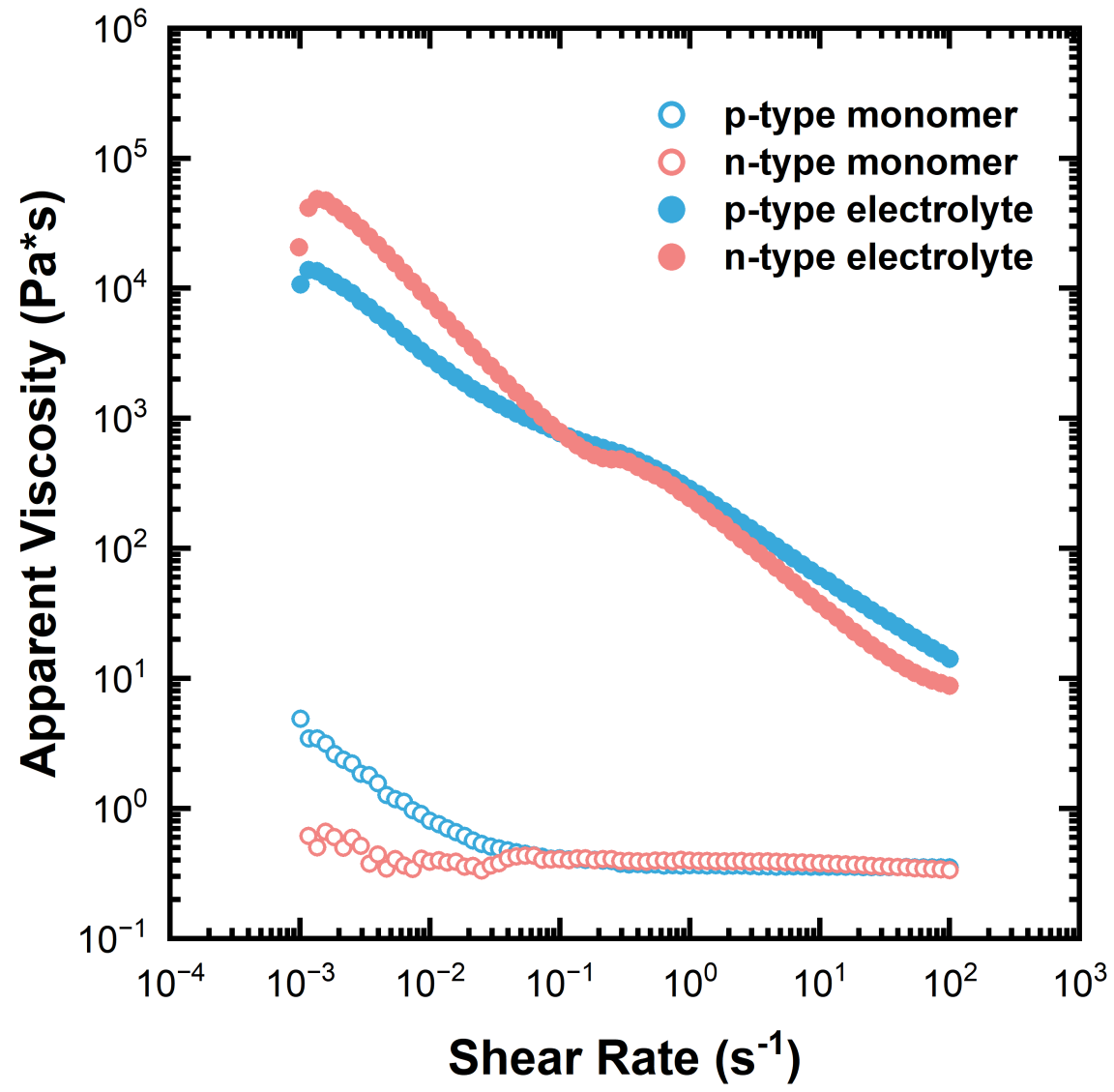

Figure S3: **Rheology of IL Monomers and Electrolyte Inks.** Flow curve for p-/n-type IL monomers and their corresponding electrolyte inks. IL monomers behave like viscous liquids, while electrolyte inks are shear thinning, yield-stress fluids.

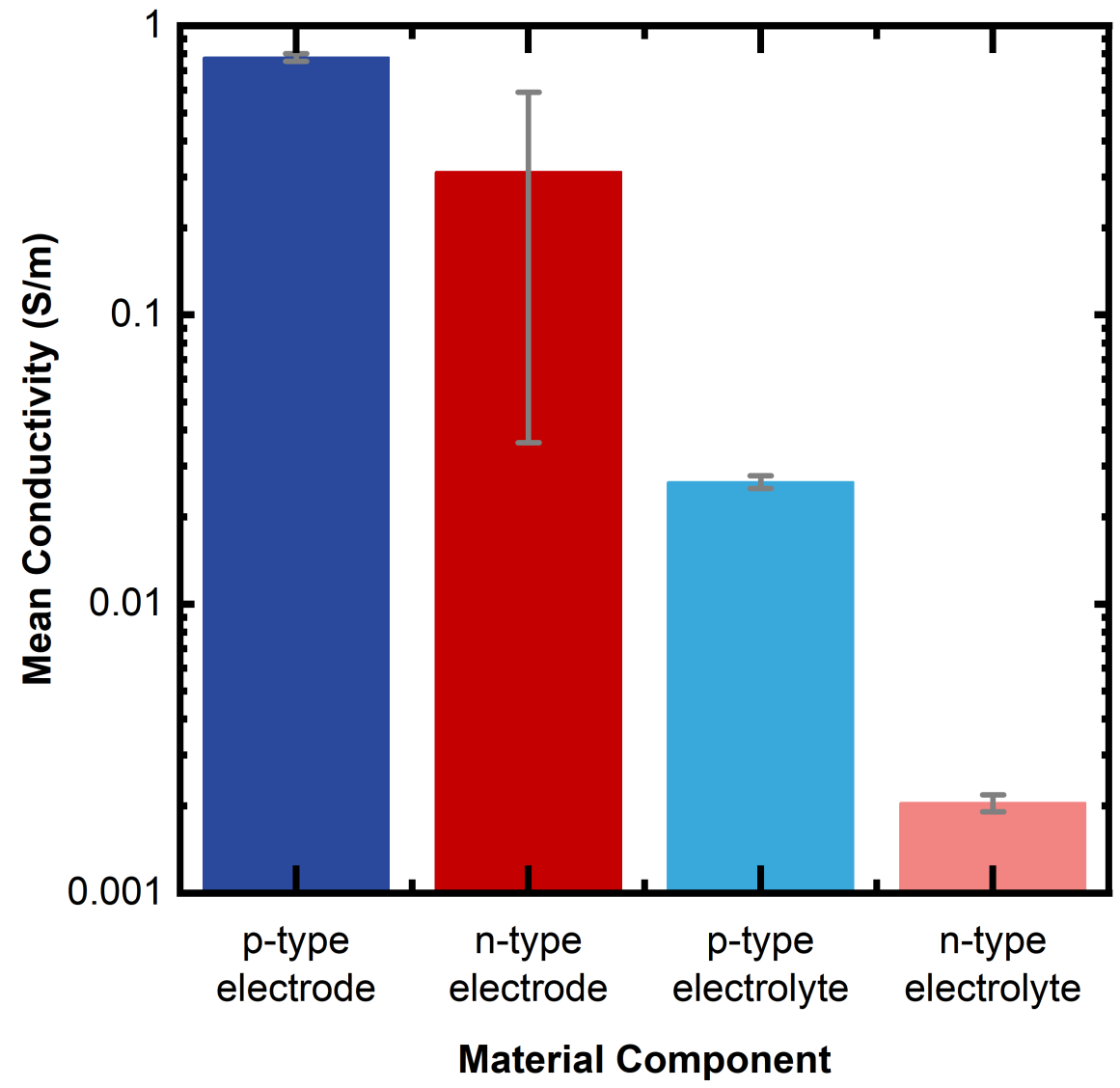

Figure S4: **Conductivity of Crosslinked Inks.** Bar plot of mean conductivity from 4-point probe for films of each material. Mixed conductor electrode materials are orders of magnitude more conductive than ionically conductive electrolytes.

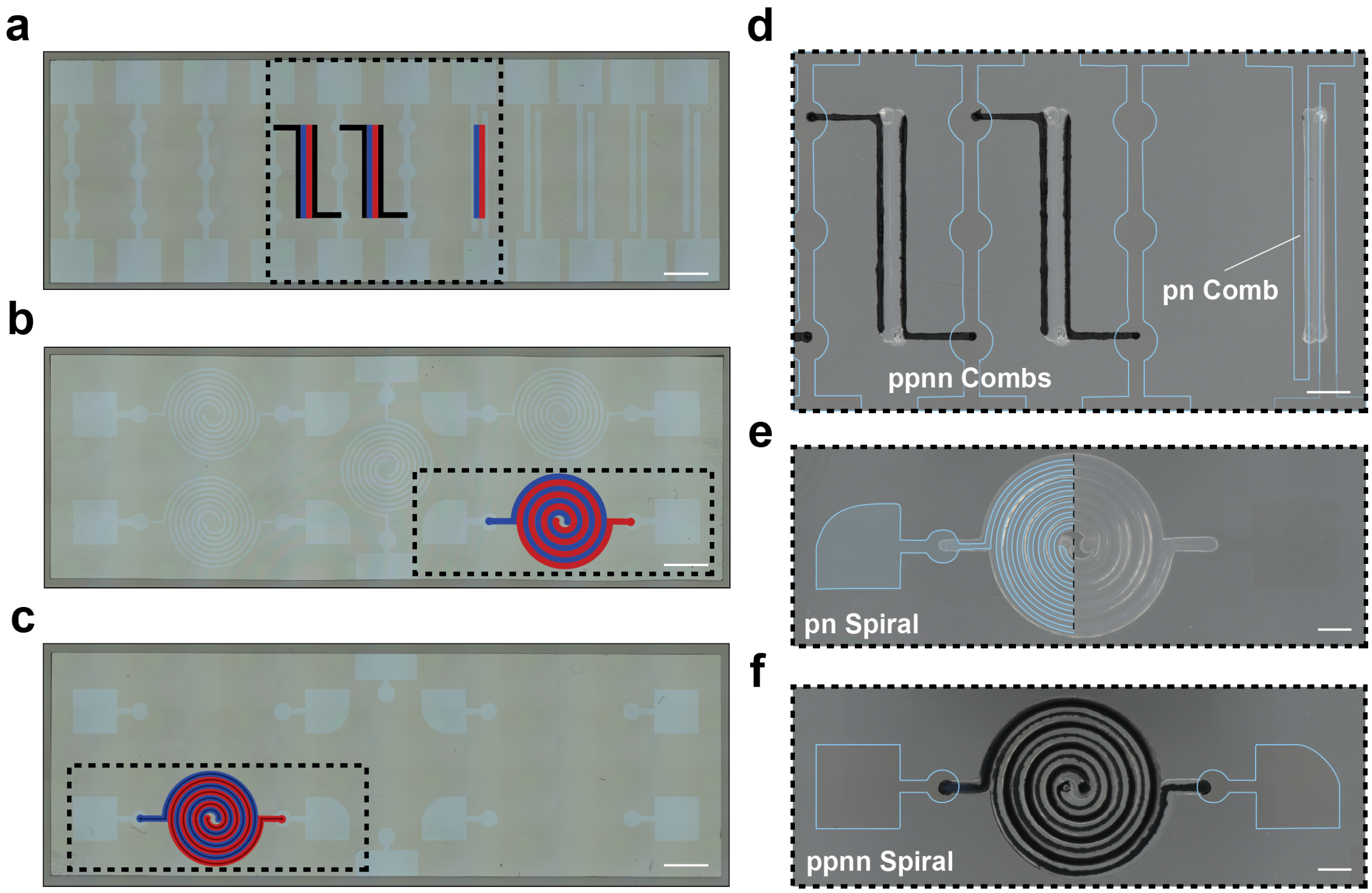

Figure S5: **Glass Substrates with Patterned ITO Electrodes.** (a-c) Optical images of ITO glass slides with pre-patterned ITO electrode features used when printing (a) pn/ppnn comb, (b) pn spiral, and (c) ppnn spiral SIDs. Each photograph is overlaid with schematic illustrations of pn/ppnn diode features as they would be aligned and printed with the ITO features. Scale bars are 5 mm. (d-f) Corresponding insets in (a) to (c) show magnified views of the glass substrates with printed (d) ppnn and pn comb, (e) pn spiral, and (f) ppnn spiral SIDs. The ITO patterns are outlined with the overlaid blue lines. Scale bars are 2 mm.

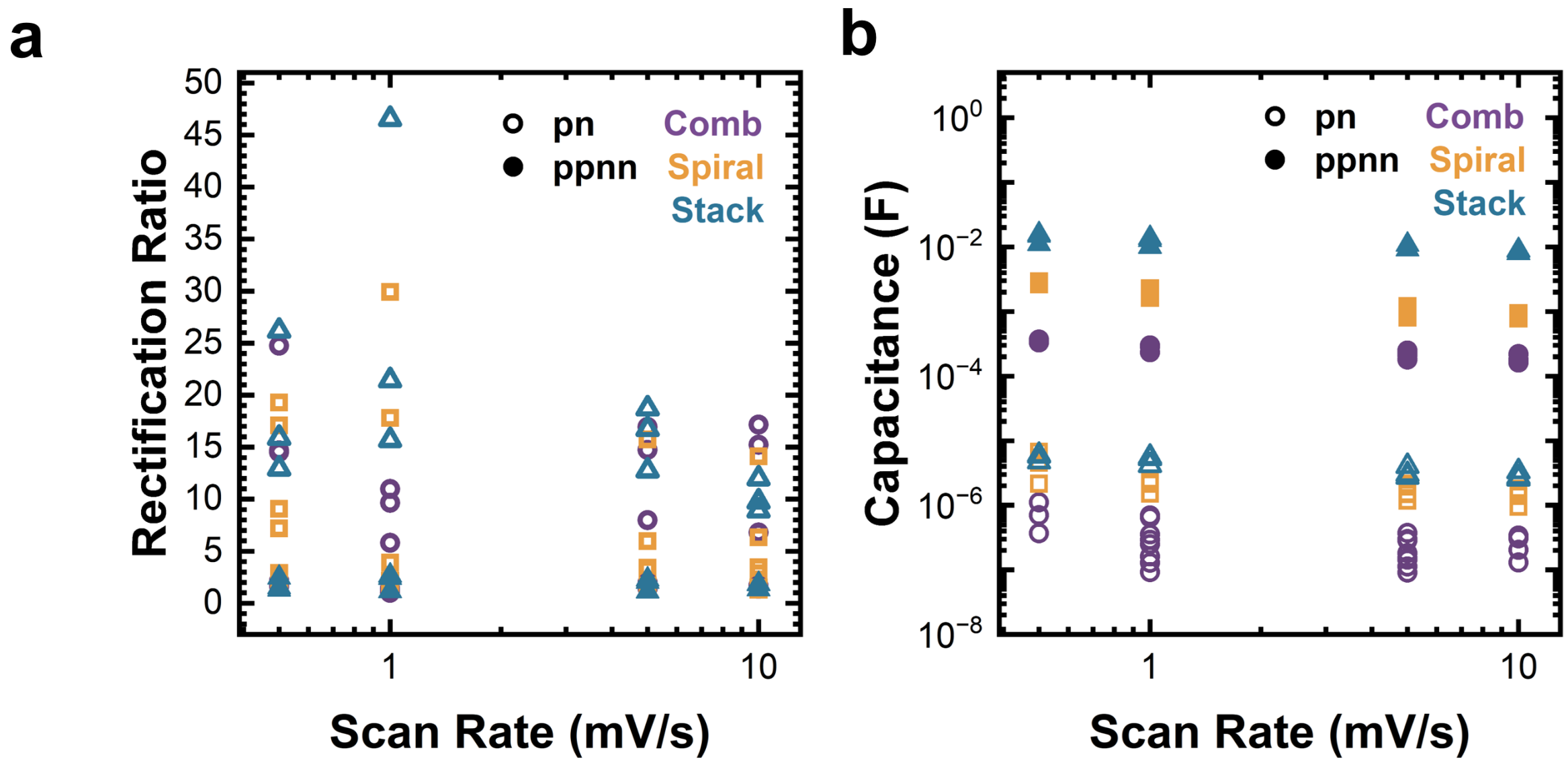

Figure S6: **Full Dataset for Figure 3.** Plots of a) rectification ratio and b) capacitance as a function of scan rate for all SIDs tested. Averages are available in Table S2.

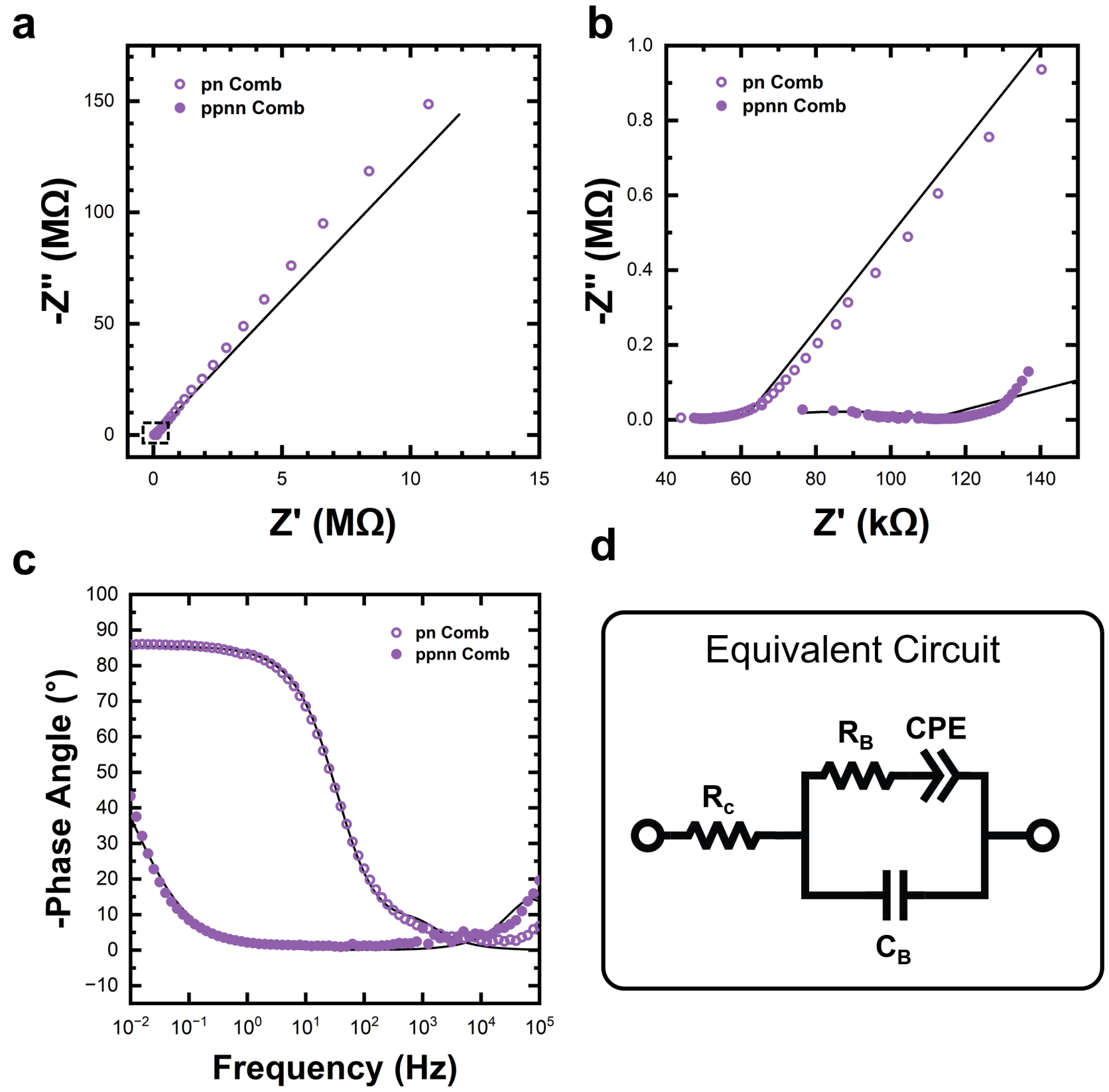

Figure S7: **Electrochemical Impedance Spectroscopy (EIS) of Comb SIDs.** (a) Nyquist plot of pn and ppnn comb SIDs, with an (b) inset region denoted with a dashed box. (c) Bode plots of pn and ppnn comb SIDs with (d) the equivalent circuit diagram. The model contains a contact resistance (R_c) and a modified RC circuit, where a dielectric capacitor accounts for bulk ionic polarization at high frequencies (C_B), and a resistor in parallel captures the the bulk resistance of free ions (R_B). A constant phase element (CPE) is added in series with the R_B resistor to describe interfacial interactions at the IDL. The impedance of a CPE is defined as $Z_{CPE}= Q_0^{-1}(j\omega)^{-\alpha}$, where Q_0^{-1} is a constant, j is the imaginary number, ω is the angular frequency, and α is a constant ranging from 0 to 1. Fitted parameters are available in Table S1.

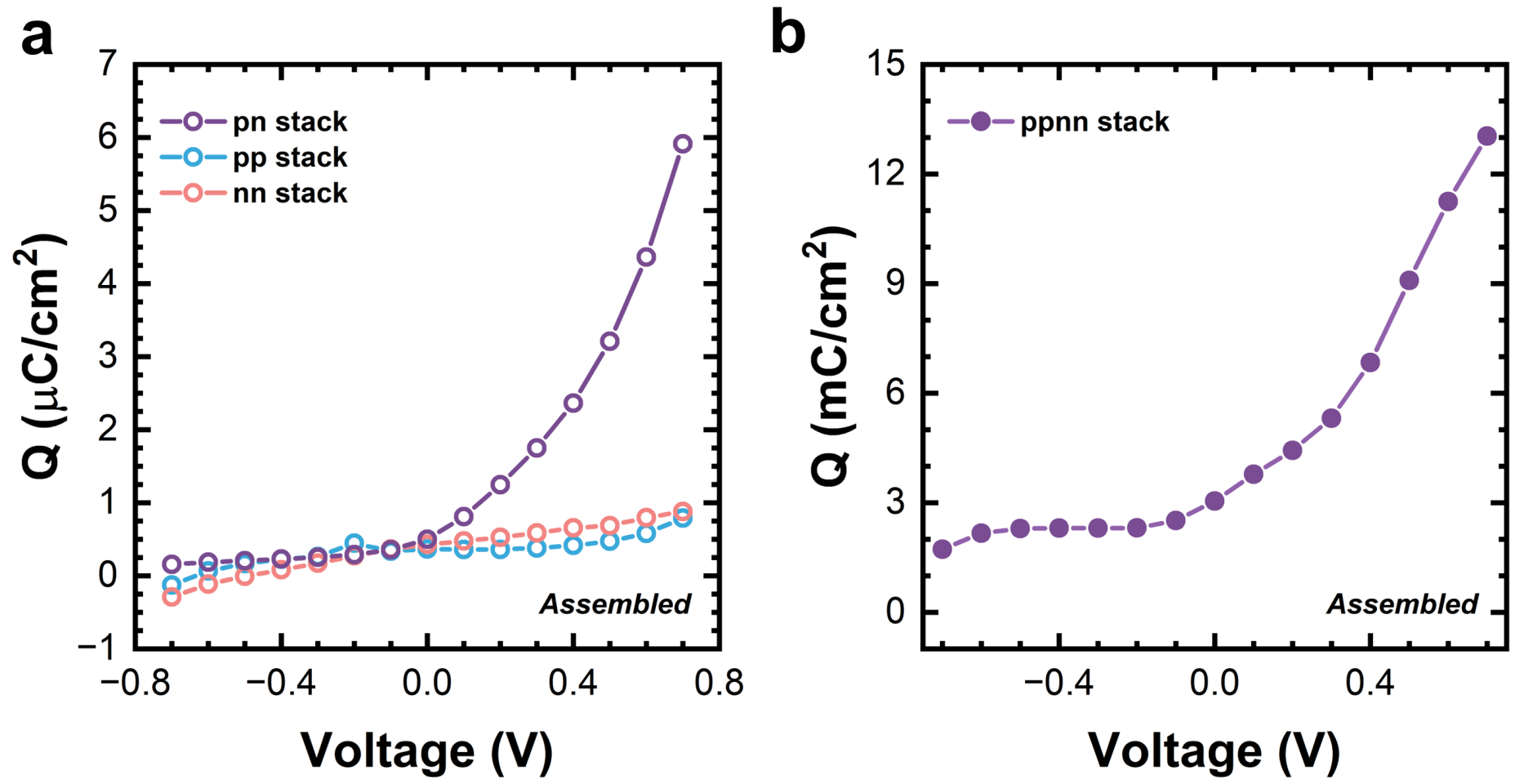

Figure S8: **Charge Accumulation in Assembled SIDs.** (a) Q-V plot for an assembled pn stacked SID and homojunctions of the p-type and n-type electrolytes. Charge accumulation was measured after 20 seconds for each voltage step. (b) Q-V plot for an assembled ppnn stacked SID, where charge accumulation was measured after 5 minutes for each voltage step.

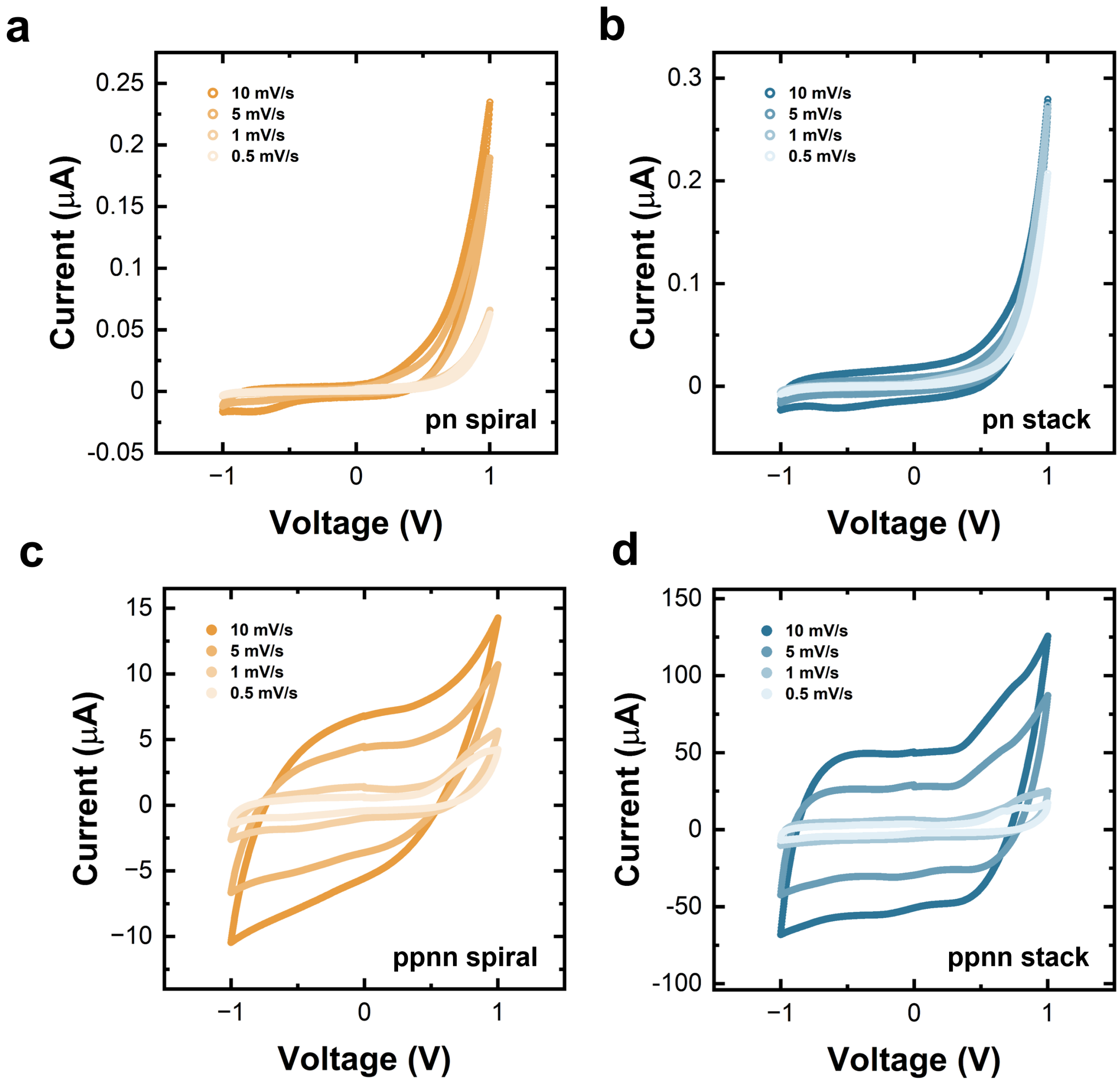

Figure S9: **Scan-rate Dependent Cyclic Voltammetry (CV) of Spiral and Stack SIDs.** CV plots for four unique (a) pn spiral, (b) pn stack, (c) ppnn spiral, and (d) ppnn stack SIDs performed at scan rates ranging from 10 mV/s to 0.5 mV/s.

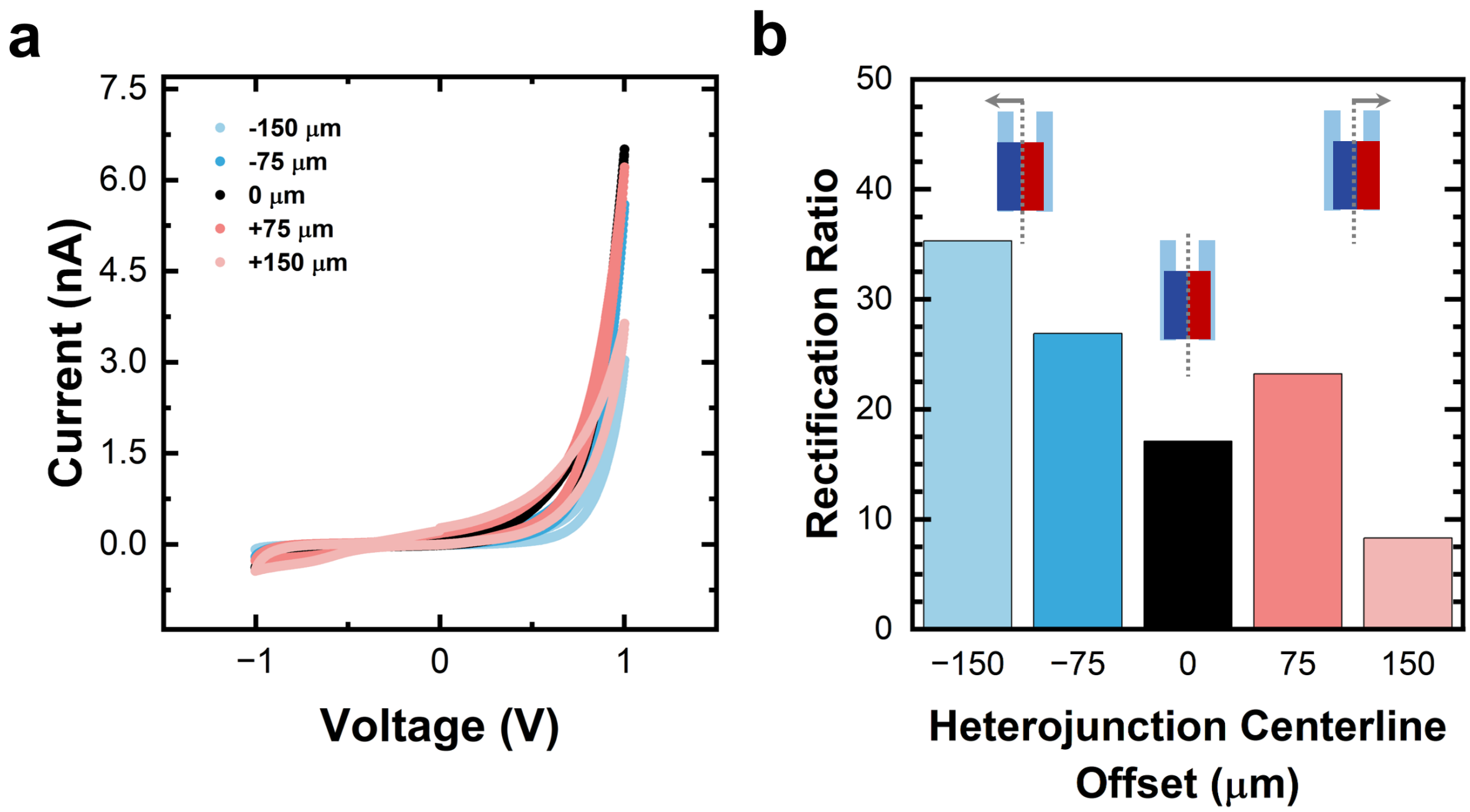

Figure S10: **Rectification Ratio Variability in 3D Printing.** Five pn comb SIDs are printed on a single substrate with pre-programmed offsets in the x-axis to shift the heterojunction towards the p-type gel (-x direction) or the towards the n-type gel (+x direction). A CV plot and the corresponding rectification ratios are depicted in (a) and (b) respectively.

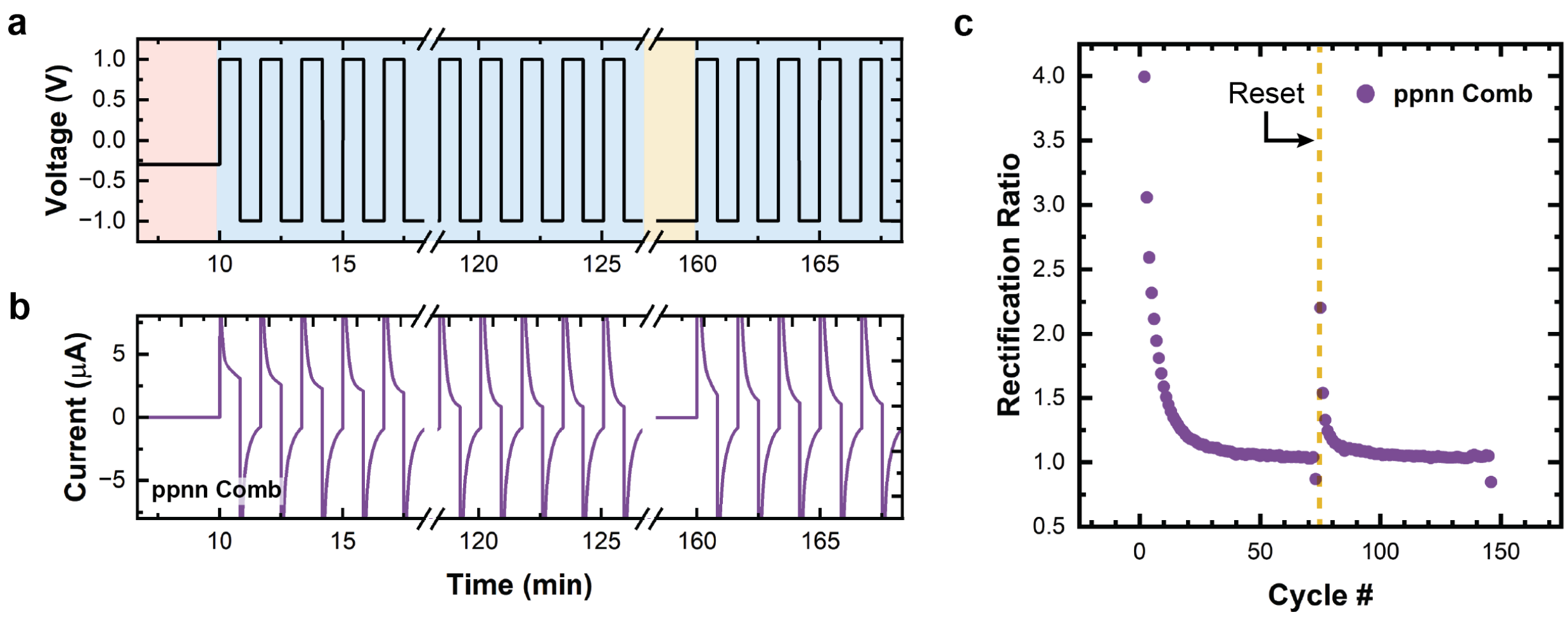

Figure S11: **Chronoamperometry of ppnn Comb SID.** (a) During a chronoamperometry (CA) experiment as outlined in Figure 4, AC voltages are applied to a ppnn comb SID (at 0.01 Hz), and (b) we record the current (I) response over time (t). (c) The corresponding change in rectification ratio of the ppnn comb SID at each cycle of the CA experiment is consistent with the performance observed in the pn comb SID in Figure 4.

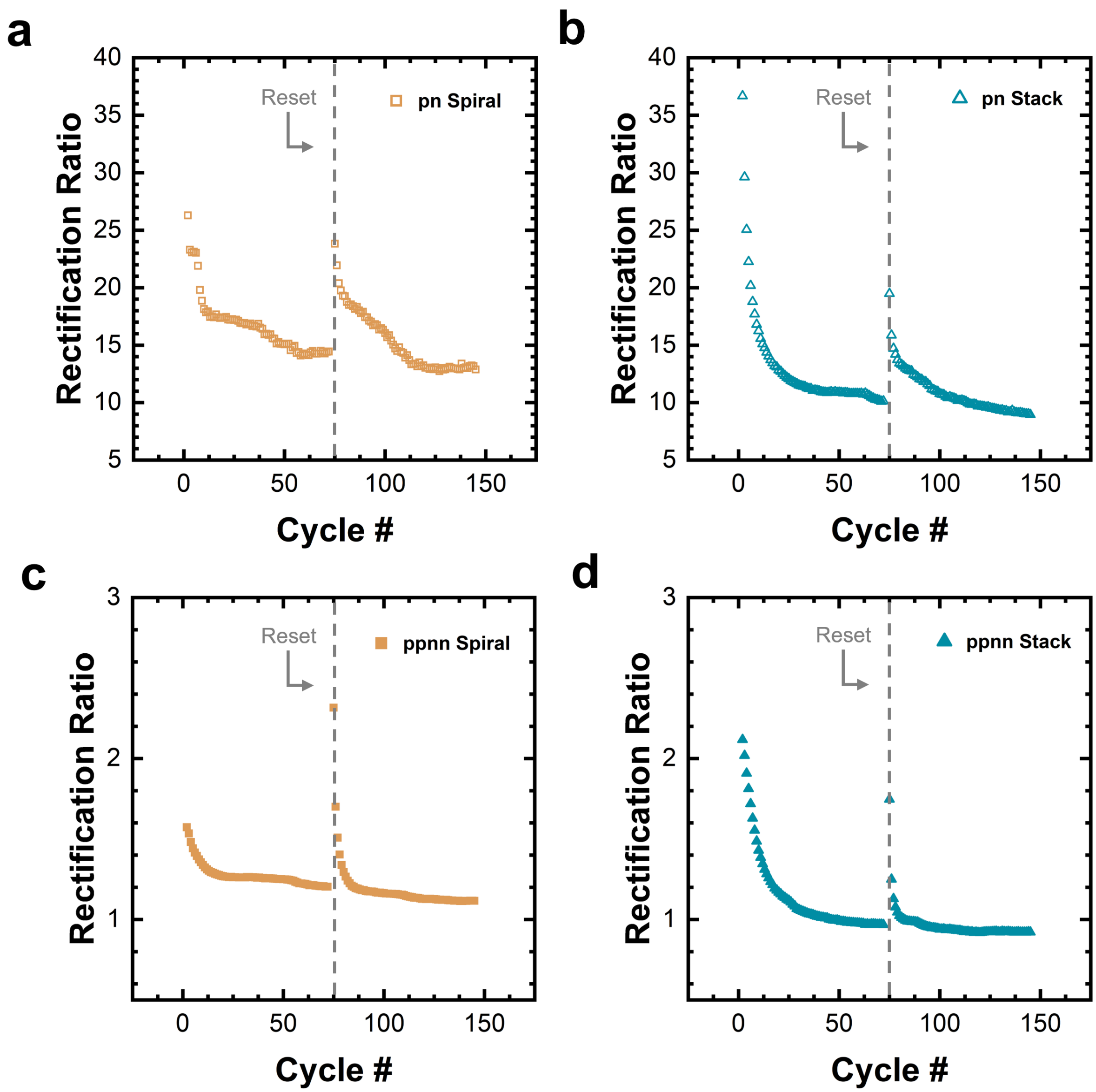

Figure S12: **Chronoamperometry of Spiral and Stack SIDs.** Spiral and stack SIDs were measured with a chronoamperometry (CA) experiment as outlined in Figure 4 (at 0.01 Hz). The corresponding rectification ratio at each cycle of the CA experiment for (a) pn spiral, (b) pn stack, (c) ppnn spiral, and (d) ppnn stack SIDs exhibit changes over time consistent with the pn comb SID shown in Figure 4.

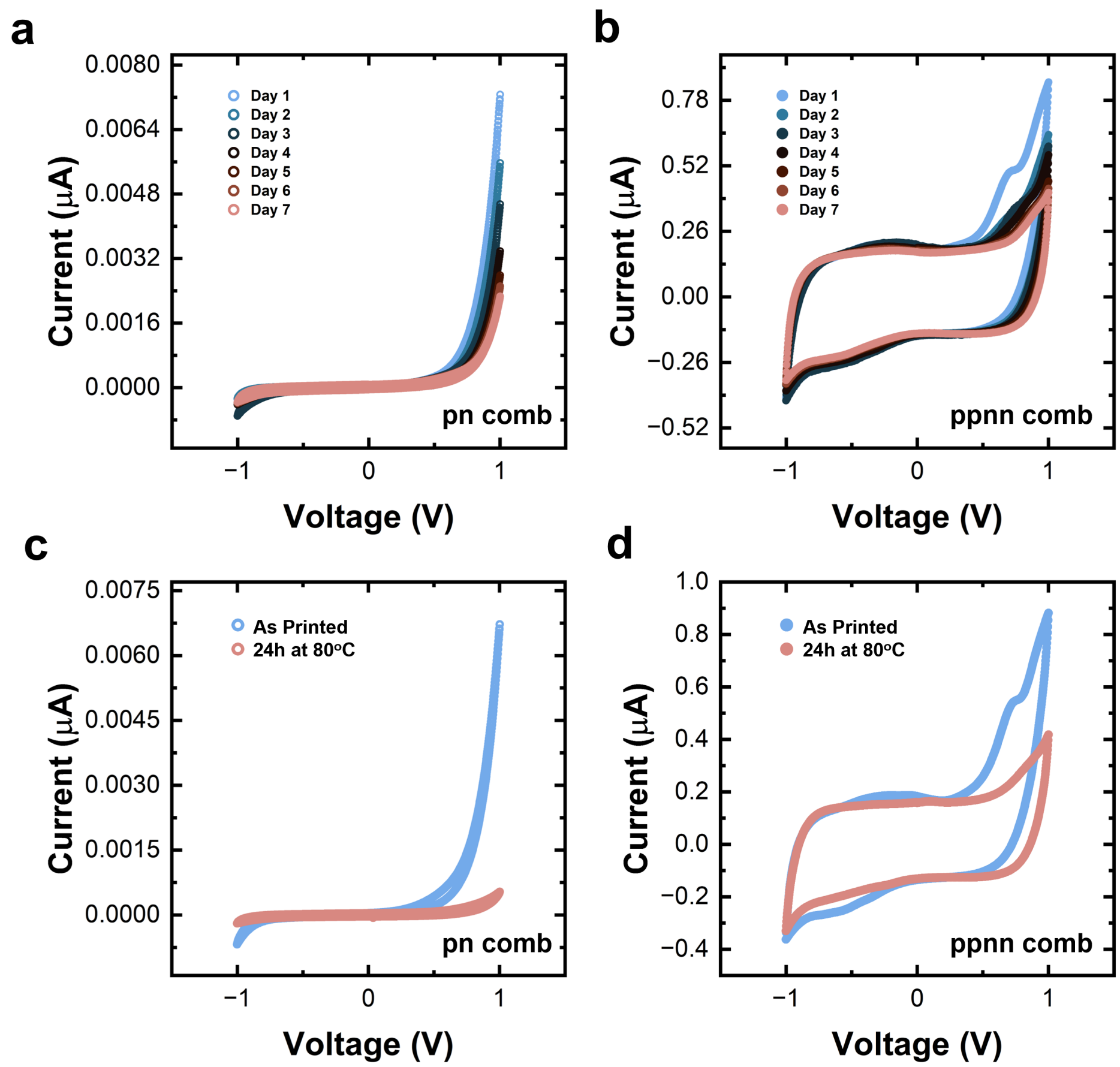

Figure S13: **Influence of Time and Temperature on SID Rectification.** Cyclic voltammetry (CV) tests on the same (a) pn comb and (b) ppnn comb SIDs taken every 24 h for one week. I_{on} decreases in each SID over time. CV tests on the same (c) pn comb and (d) ppnn comb SIDs after heating the printed SIDs at 80°C for 24 h. Rectification after heating is minimal.

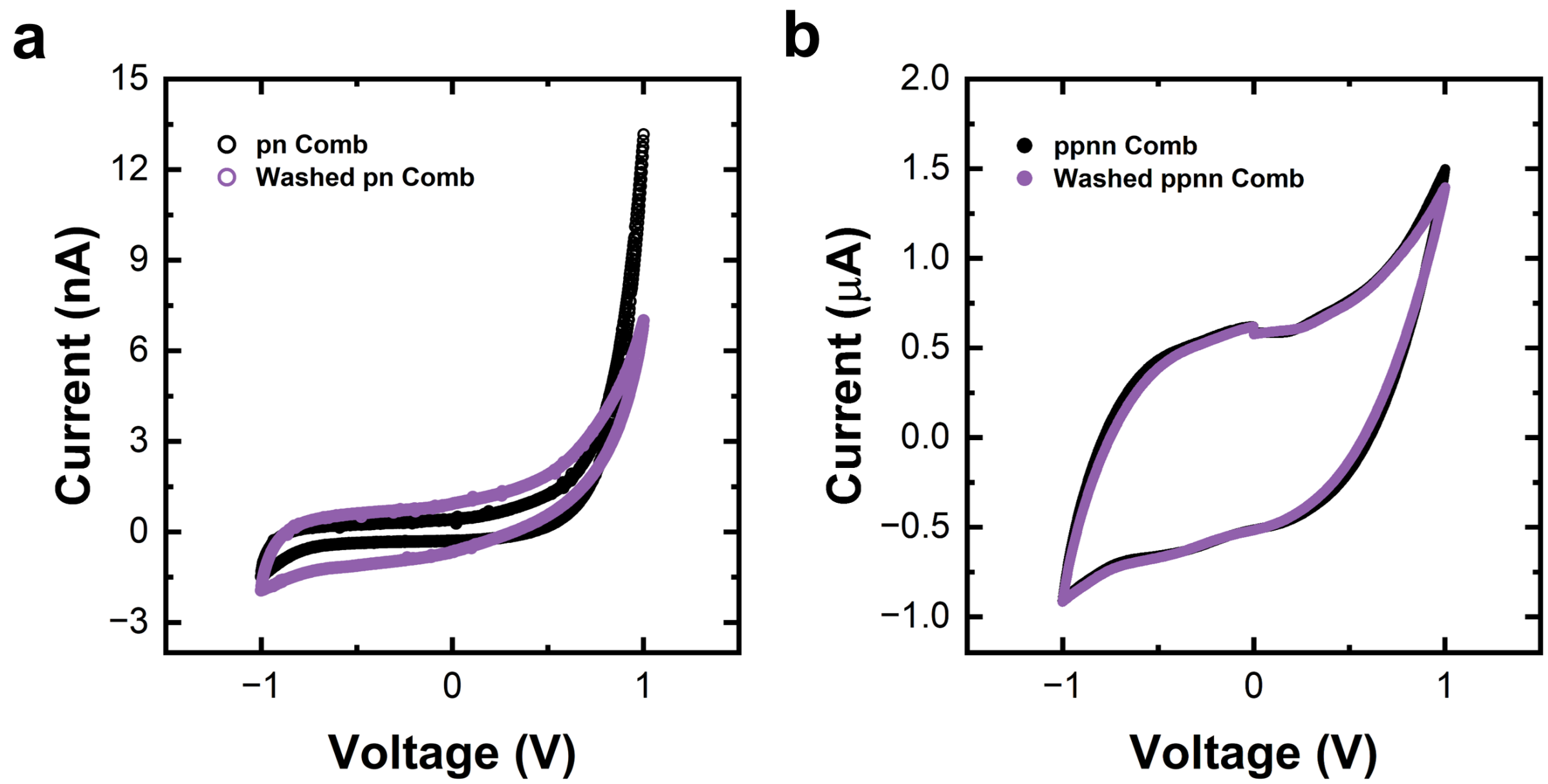

Figure S14: **Rectification in Washed SIDs.** CV plots of (a) pn comb and (b) ppnn comb SIDs before (black) and after (purple) washing cured SIDs with dichloromethane (DCM). SIDs were cured and then rinsed by pipetting aliquots of DCM over an angled substrate for 2 minutes. DCM was allowed to evaporate in a 60°C oven for 30 minutes prior to measuring CV. For both structures, the rectification ratio decreased.

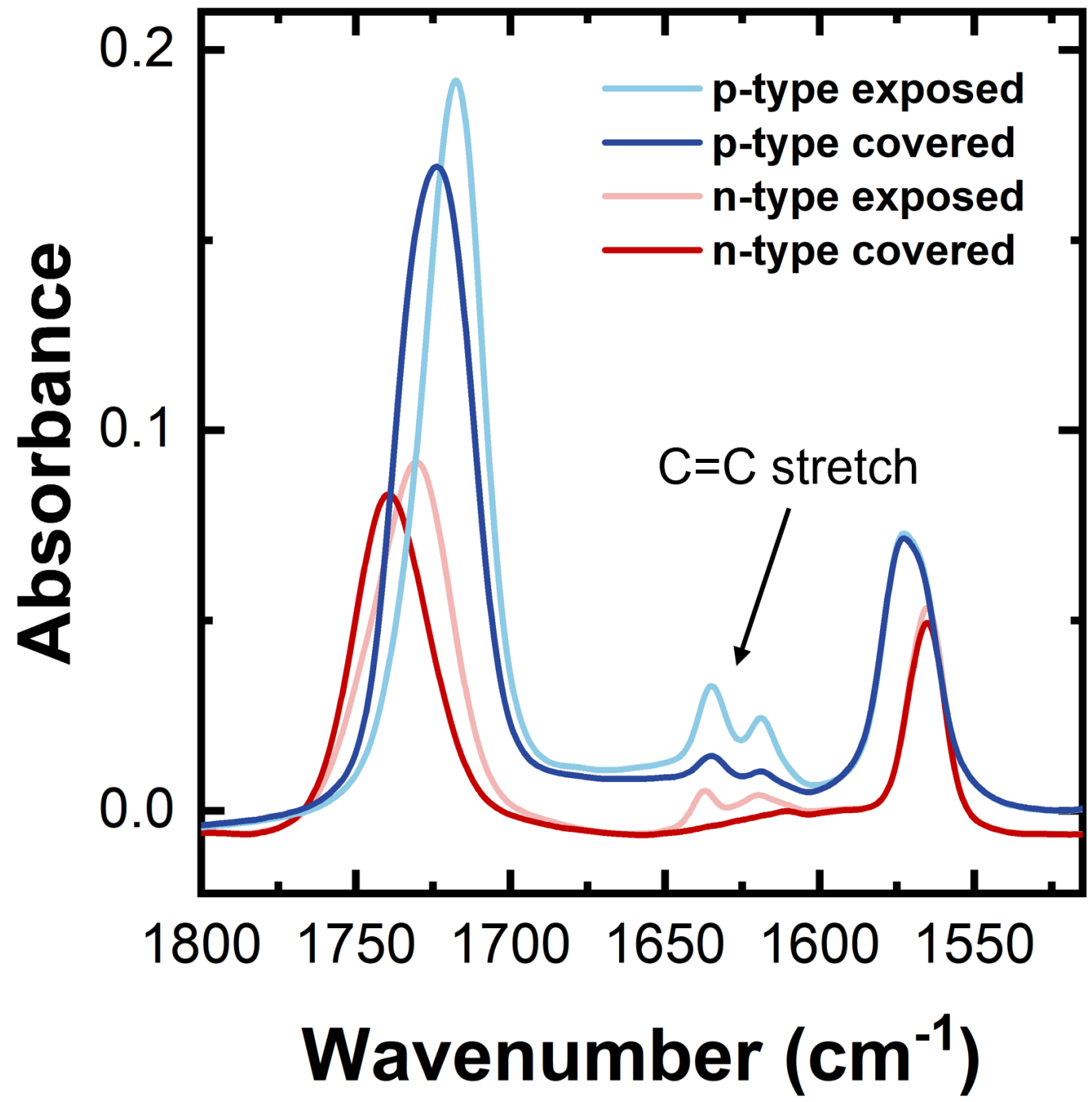

Figure S15: **ATR-FTIR of p-/n-type Electrolyte Inks.** Plot of wavenumber versus absorbance for p-/n-type electrolyte inks that were photocrosslinked under ambient air (i.e., exposed conditions) and sandwiched between glass slides (i.e., covered to minimize oxygen inhibition). The FTIR data shows residual monomers exist near the surface of photocrosslinked electrolyte inks prepared via both methods, especially in inks exposed to ambient air.

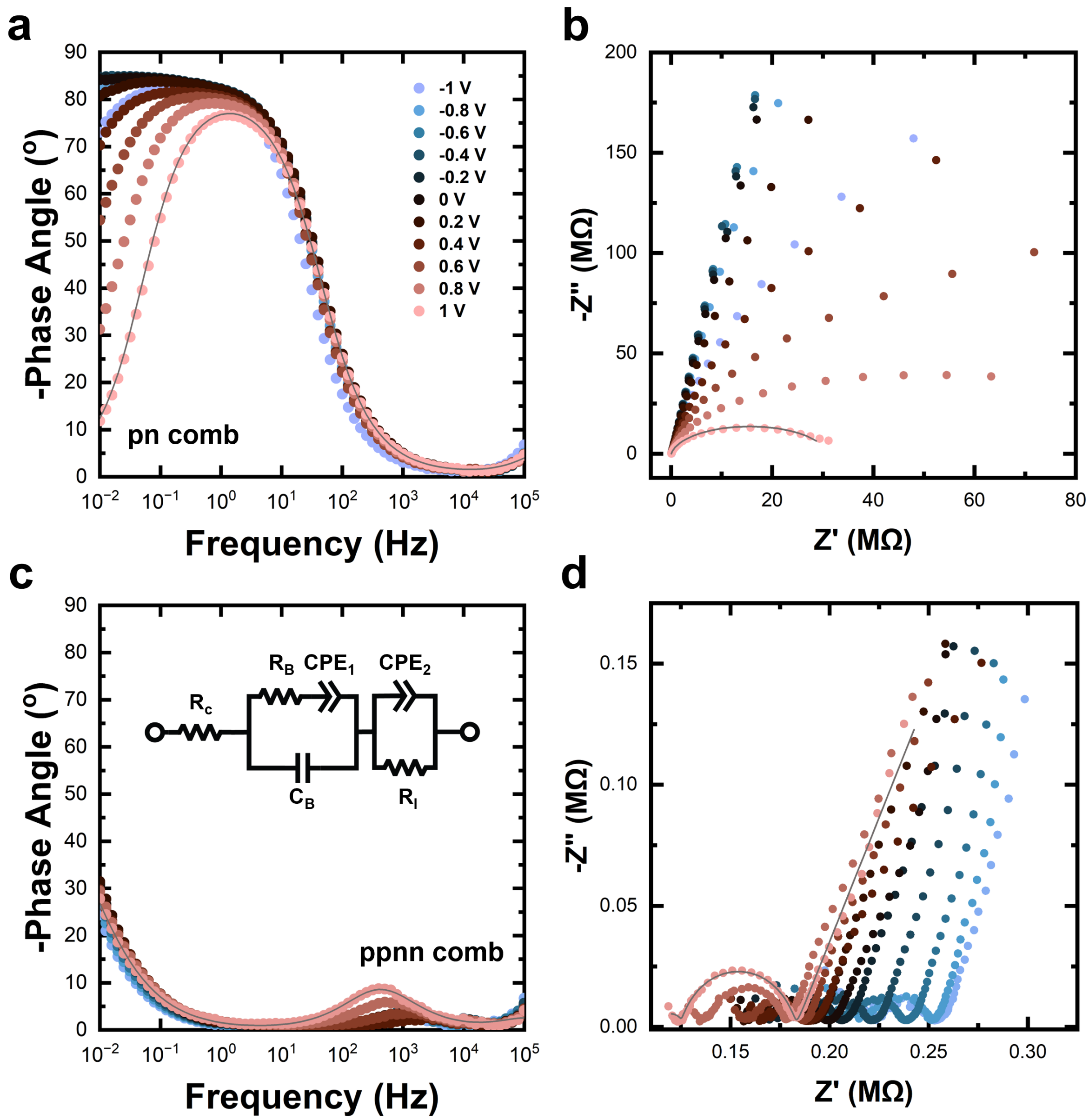

Figure S16: **EIS with Applied DC Bias of Comb SIDs.** (a) Bode and (b) Nyquist plots of a printed pn comb SID, where a DC bias is applied for 5 minutes prior to beginning the EIS experiment centered around the bias voltage. (c) Bode and (d) Nyquist plots for a printed ppnn comb SID under the same experimental conditions. Data for 1 V for both printed SIDs are fitted to the equivalent circuit model depicted in (c). The model extends the circuit in Figure S7 by adding a second modified RC circuit in series with the original circuit. The new CPE element accounts for the expected contribution of IDL capacitance (CPE_2) and is in parallel with a resistor that measures the interfacial resistance across the heterojunction (R_I). CPE_1 describes the expected capacitance of the EDL interfaces. Fitted parameters are available in Table S3.

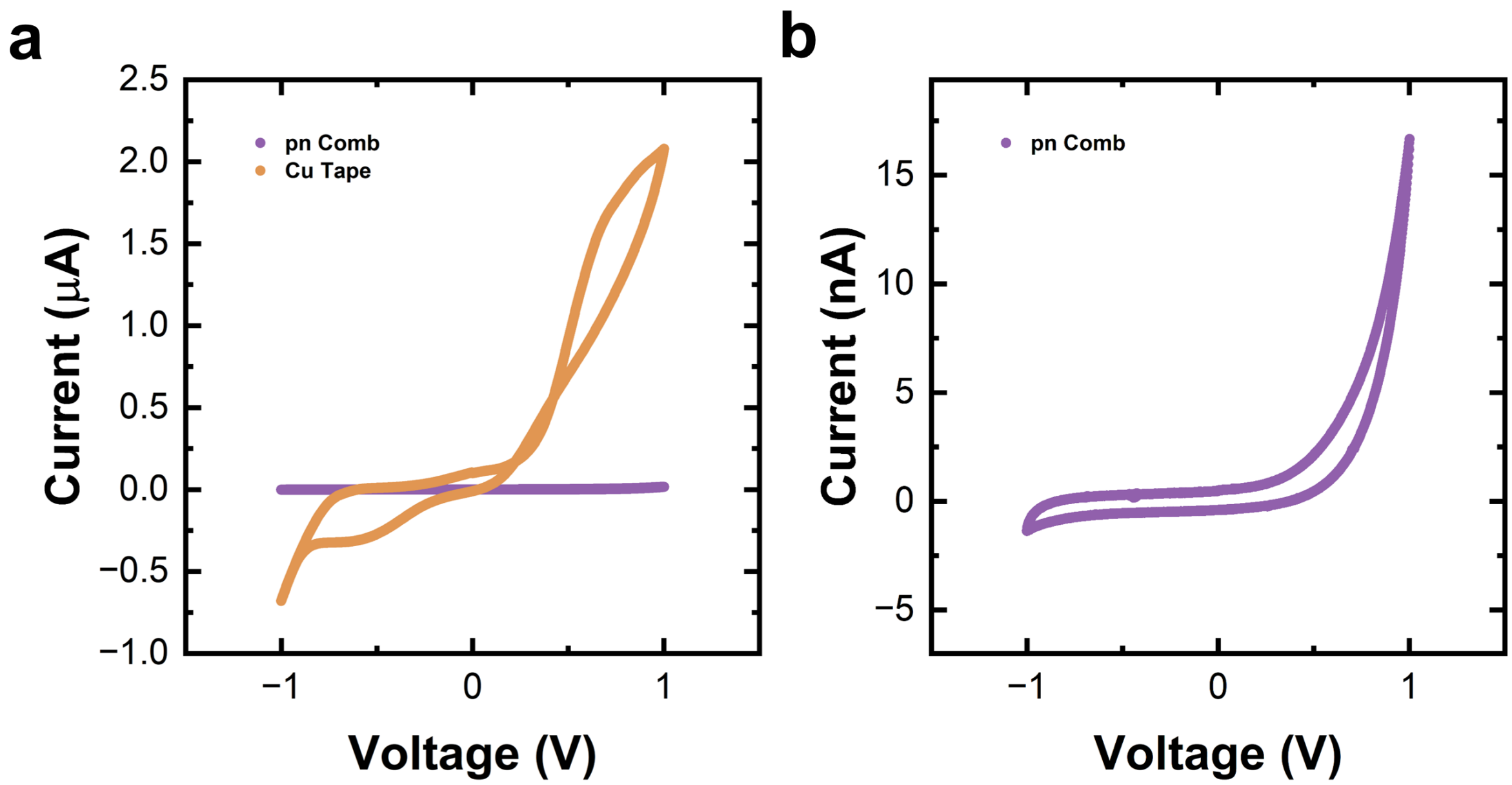

Figure S17: **Redox-active Electrodes in SIDs.** (a) CV plot of a printed pn comb SID on patterned ITO glass (purple, inset in (b)) and on conductive copper tape (yellow). Significant redox-activity between the SID and copper tape occurs, dramatically increasing the current in both the forward and reverse bias directions and decreasing the rectification ratio.

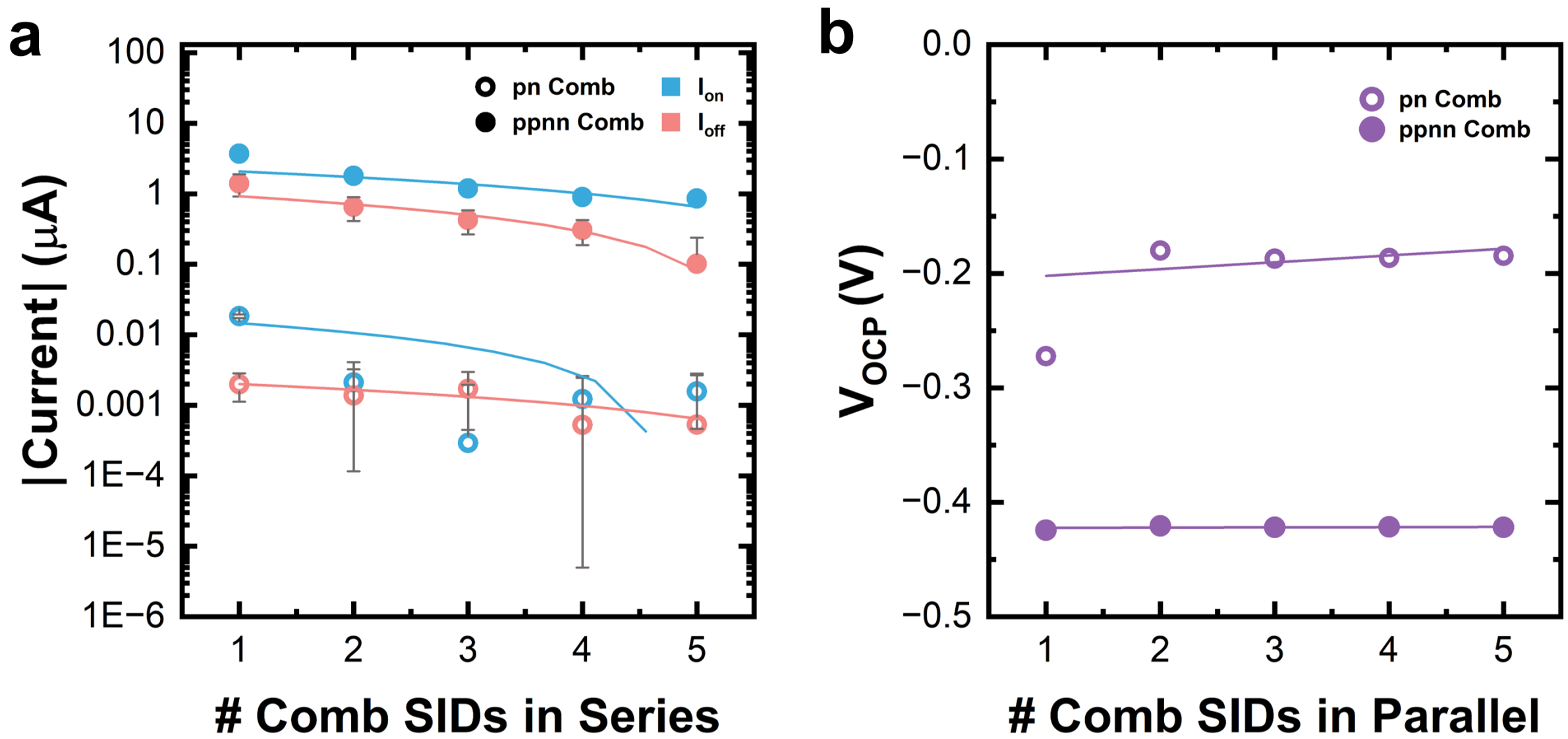

Figure S18: **Multi-SID Circuit Controls.** (a) Plot of I_{on} and I_{off} versus number of comb SIDs in series shows a decrease in current with additional SIDs. The current values are taken from CA data at $V_{\text{pk}} = \pm 1$ V and $f = 0.1$ Hz, averaged across the first 5 cycles. Error bars have been removed when the error is smaller than the plotted point. (b) Plot of open circuit voltage (V_{OCP}) versus number of comb SIDs in parallel shows little change in V_{OCP} for additional SIDs. V_{OCP} is averaged across 10 s, and the error is smaller than the plotted points. The trend lines shown in (a) and (b) are linear fits of the corresponding data.

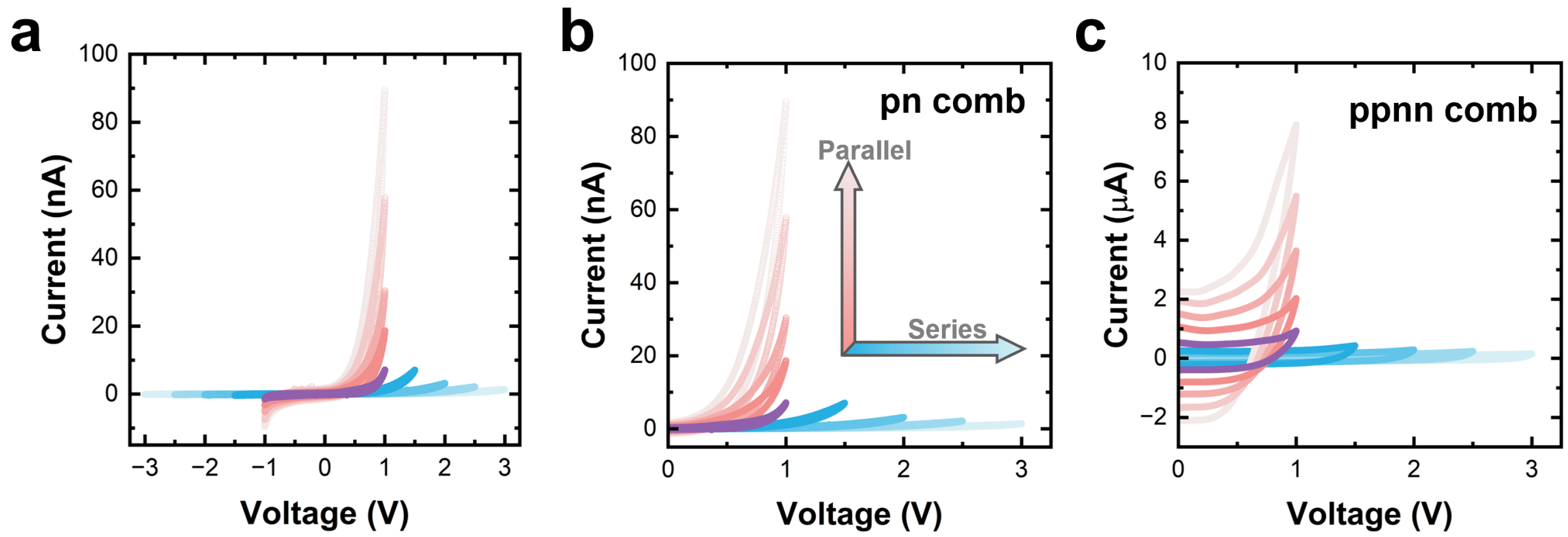

Figure S19: **Multi-SID Circuit CVs.** (a) CV plot of printed pn comb SID arrays combined in parallel (pink gradient) and series (blue gradient). CV plots showing only the forward bias response of printed (b) pn comb and (c) ppnn comb SID arrays. As comb SIDs are added in parallel, the effective heterojunction interface area increases, increasing the net current and capacitance that are measured. Comb SIDs added in series broaden the voltage window of the multi-SID circuit before rectification occurs.

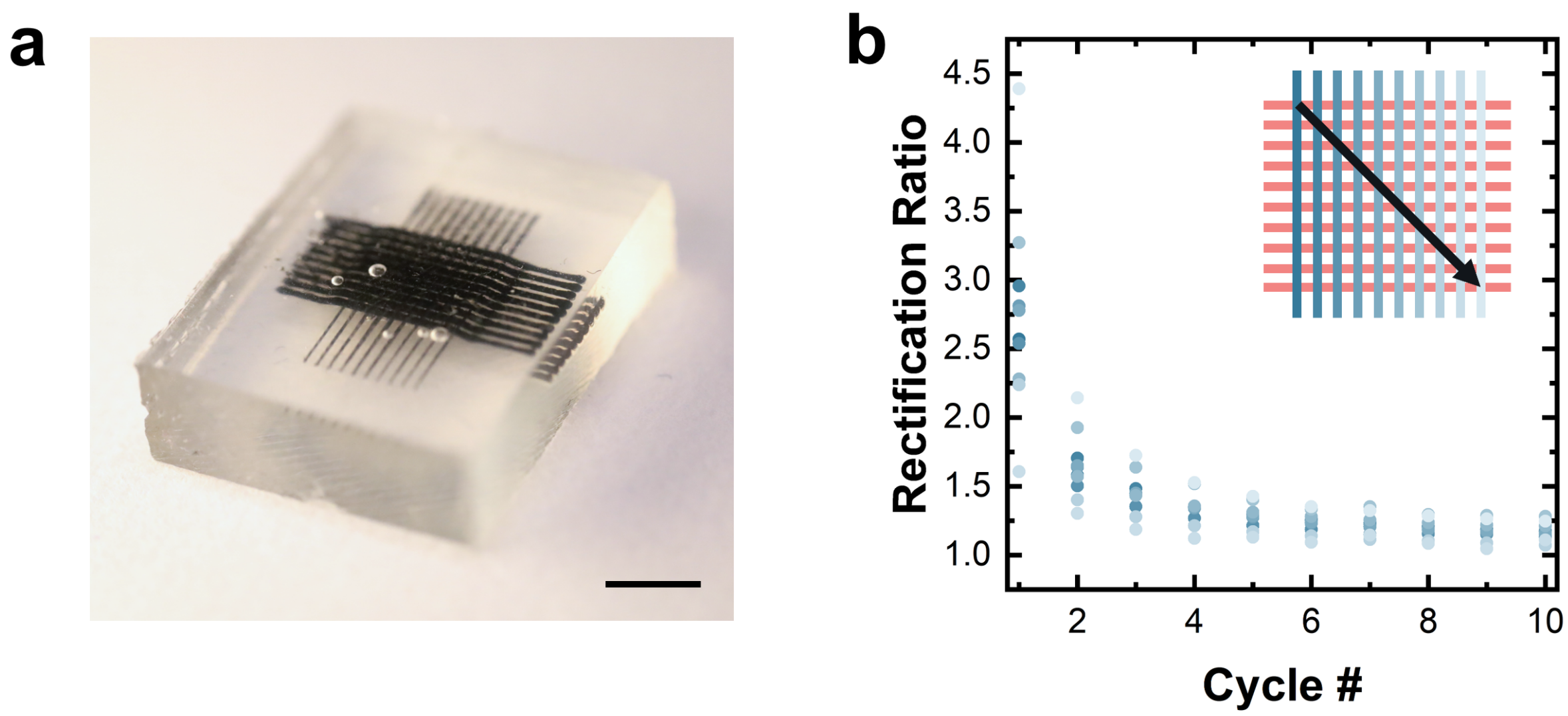

Figure S20: **Multi-SID Crossbar Array.** (a) Photograph of a printed multi-SID array encased in silicone elastomer. Scale bar is 5 mm. (b) Plot of rectification ratio at each cycle of a CA experiment (0.016 Hz, or 1 cycle/min) for 10 unique SID intersections. SIDs were measured along the diagonal as depicted in the inset schematic. The rectification ratio for each SID is unique but consistent with the range of values expected for stacked SIDs.

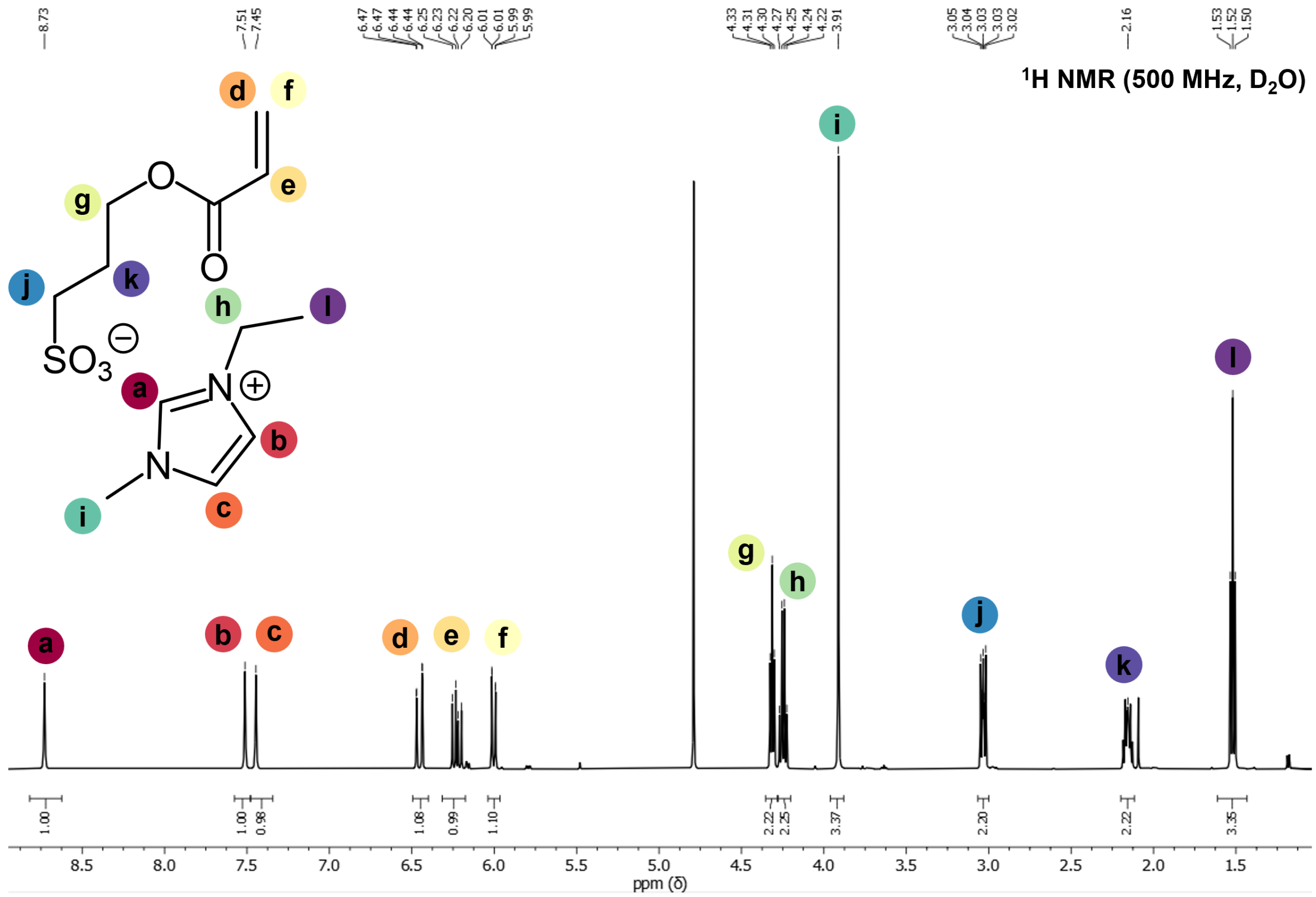

Figure S21: **^{1}H NMR of p-type IL monomer.** 1-ethyl-3-methylimidazolium (3-sulfopropyl)acrylate (ES) chemical structure and ^{1}H NMR (500 MHz, D_2O): δ 8.73 (s, 1H), 7.51 (s, 1H), 7.45 (s, 1H), 6.45 (dd, 1H, $J = 17.4, 1.1$ Hz), 6.23 (dd, 1H, $J = 17.3, 10.5$ Hz), 6.00 (dd, 1H, $J = 10.5, 1.1$ Hz), 4.31 (t, 2H, $J = 6.3$ Hz), 4.25 (q, 2H, $J = 7.3$ Hz), 3.91 (s, 3H), 3.06-3.01 (m, 2H), 2.20-2.12 (m, 2H), 1.52 (t, 3H, $J = 7.4$ Hz).

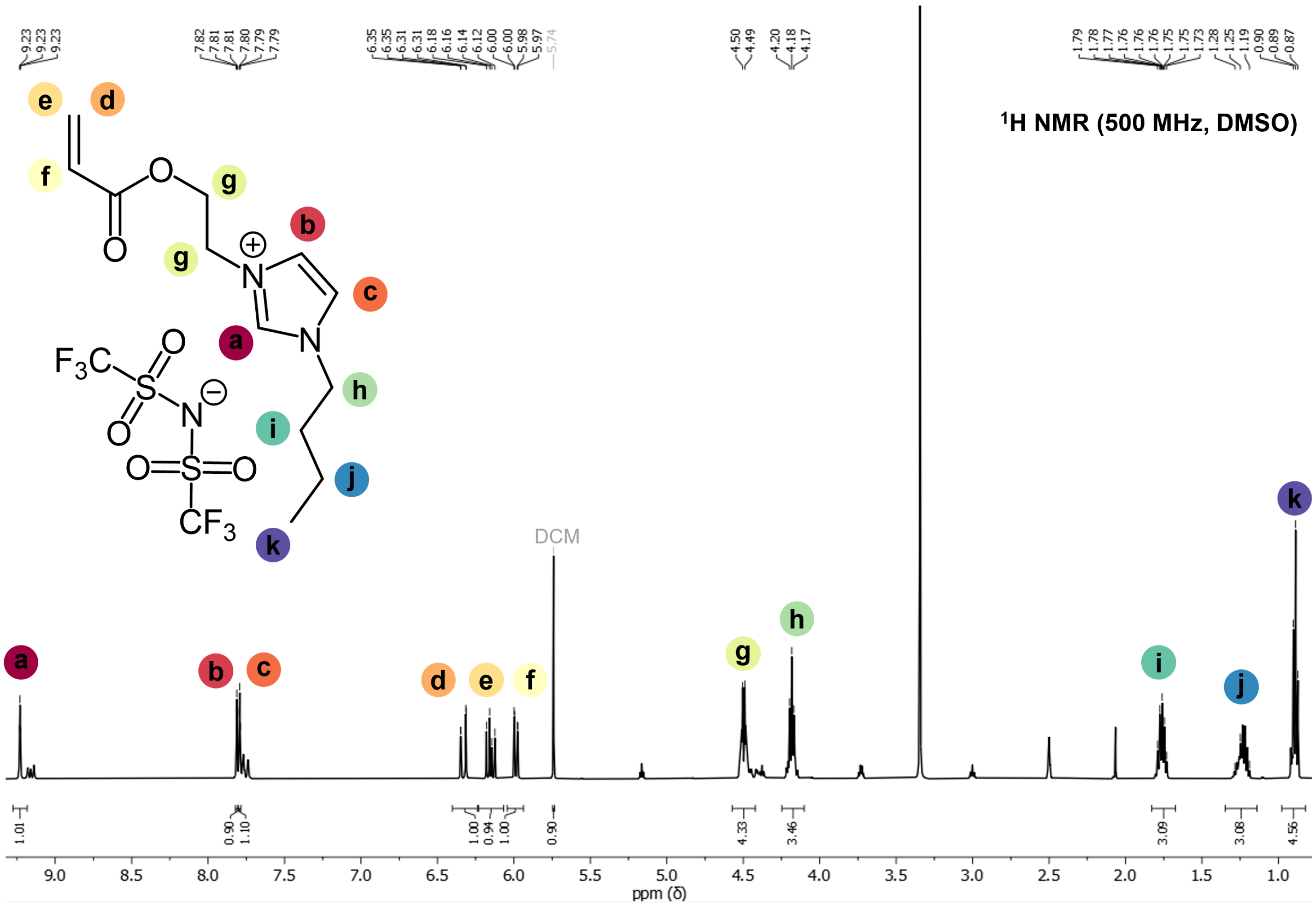

Figure S22: **^{1}H NMR of n-type IL monomer.** 1–[2–acryloyloxyethyl]–3–butylimidazolium bis(trifluoromethane)sulfonimide (AT) chemical structure and ^{1}H NMR (500 MHz, DMSO): δ 9.23 (t, 1H, $J = 1.6$ Hz), 7.81 (t, 1H, $J = 1.8$ Hz), 7.79 (t, 1H, $J = 1.8$ Hz), 6.33 (dd, 1H, $J = 17.3, 1.4$ Hz), 6.15 (dd, 1H, $J = 17.3, 10.4$ Hz), 5.99 (dd, 1H, $J = 10.4, 1.4$ Hz), 4.53 – 4.47 (m, 4H), 4.18 (t, 3H, $J = 7.1$ Hz), 1.82 – 1.71 (m, 2H), 1.30 – 1.18 (m, 2H), 0.89 (t, 3H, $J = 7.4$ Hz).

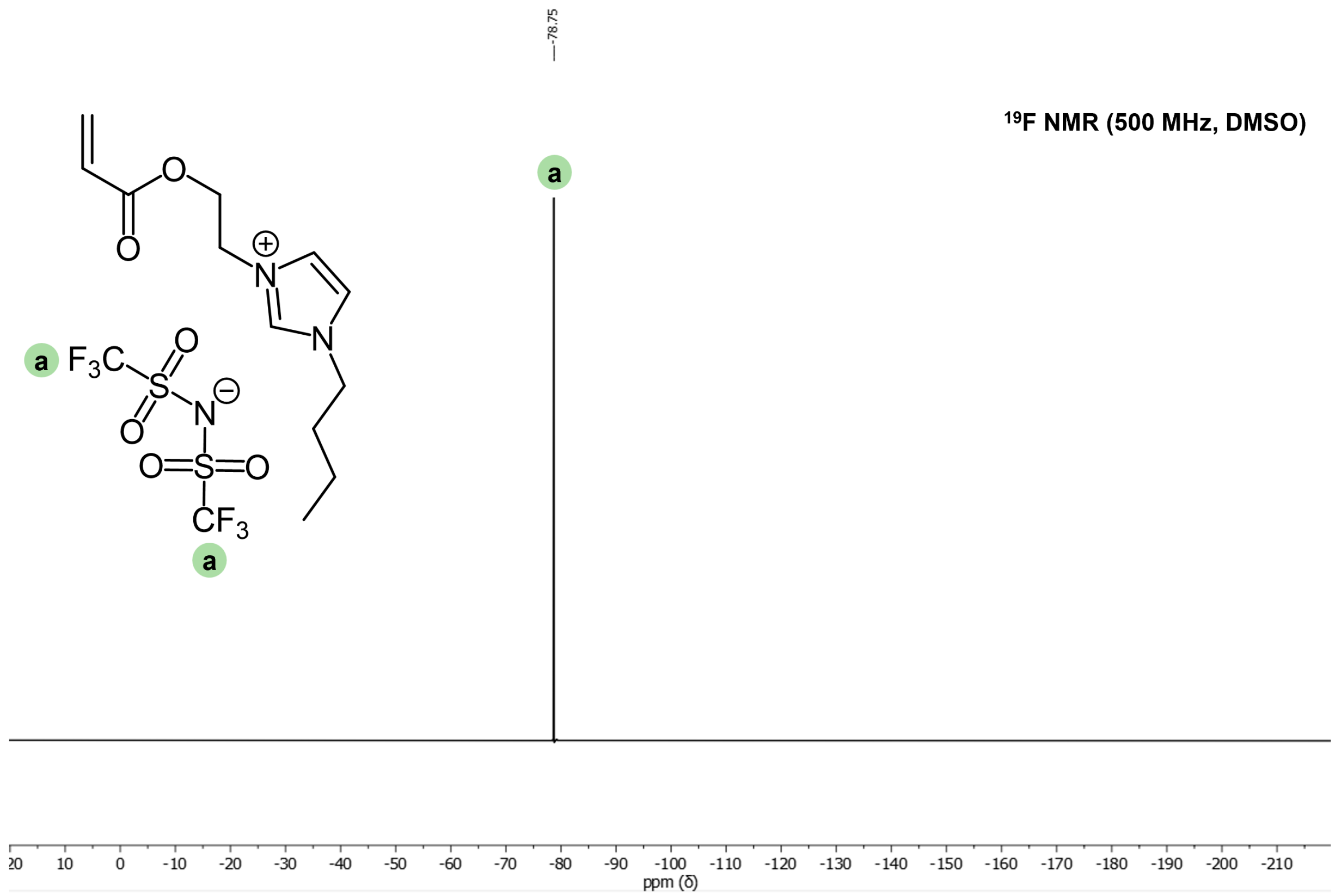

Figure S23: **^{19}F NMR of n-type IL monomer.** 1–[2–acryloyloxyethyl]–3–butylimidazolium bis(trifluoromethane)sulfonimide (AT) chemical structure and ^{19}F NMR (500 MHz, DMSO): δ -78.8 (s, 6F).

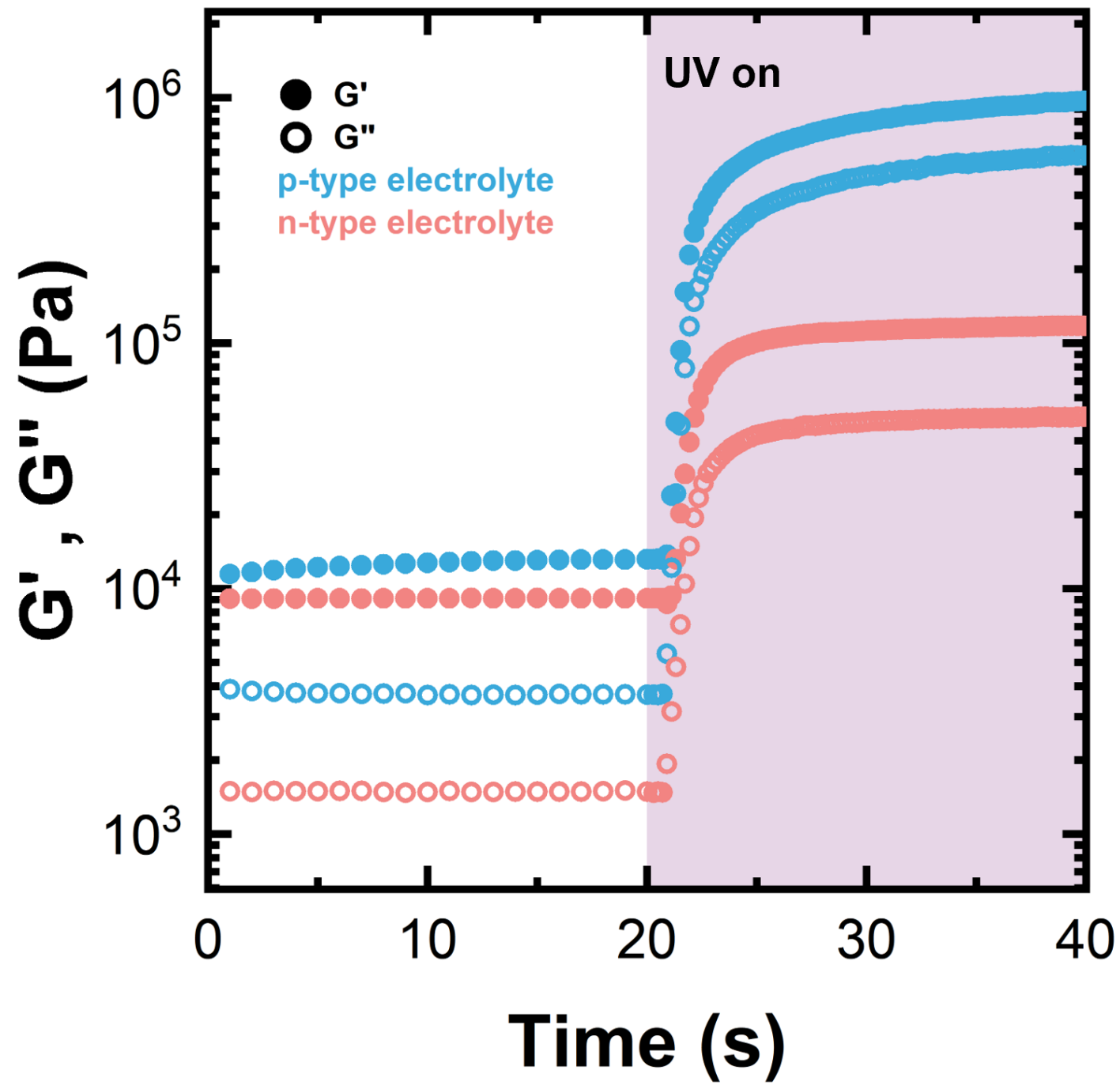

Figure S24: **Photorheology of Electrolyte Inks.** Plot of storage (G') and loss (G") moduli versus shear stress for electrolyte inks before and after UV exposure (1 W/cm^2) during an oscillatory experiment (10 Hz).

Table S1: **EIS Fitting Parameters from Figure S7**.

Printed Structure	R_C (kΩ)	R_B (kΩ)	CPE		C_B (pF)	Area (cm^2)	C_{eff} (μF/cm^2)
			Q_0	α			
**pn Comb**	49.7 ± 0.4	18.4 ± 1.6	8.02 ± 0.19 x 10^{-9}	0.94 ± 0.01	15.4 ± 1.8	0.025	2.10
**ppnn Comb**	66.3 ± 4.6	43.3 ± 4.5	6.62 ± 0.50 x 10^{-5}	0.77 ± 0.03	63 ± 15	0.025	3650

Note: Fitted parameters are defined with respect to the equivalent circuit in Figure S7. C_{eff} is calculated from the Brug model, where $C_{\text{eff}} = Q_0^{1/\alpha} R_B^{1/\alpha - 1}$

Table S2: **Extended Dataset with Error for Figure 3.**

Metric	Scan Rate (mV/s)	pn Comb	pn Spiral	pn Stack
**Rectification Ratio**	0.5	18.0 ± 5.6	11.8 ± 6.5	18.3 ± 7.0
	1	8.8 ± 2.7	17.2 ± 13.0	27.9 ± 16.4
	5	13.2 ± 4.7	8.4 ± 6.5	16.1 ± 3.0
	10	13.1 ± 5.5	8.0 ± 5.5	10.2 ± 1.6
**Capacitance (μF)**	0.5	0.7 ± 0.4	4.5 ± 1.9	5.5 ± 0.7
	1	0.3 ± 0.2	2.0 ± 0.4	5.0 ± 0.7
	5	0.2 ± 0.1	1.6 ± 0.4	3.3 ± 0.7
	10	0.2 ± 0.1	1.4 ± 0.4	2.9 ± 0.4
**Metric**	**Scan Rate (mV/s)**	**ppnn Comb**	**ppnn Spiral**	**ppnn Stack**
**Rectification Ratio**	0.5	2.0 ± 0.3	2.3 ± 0.7	1.8 ± 0.6
	1	1.5 ± 0.6	1.9 ± 0.2	2.1 ± 0.8
	5	1.7 ± 0.3	1.5 ± 0.2	1.8 ± 0.6
	10	1.5 ± 0.2	1.4 ± 0.1	1.7 ± 0.3
**Capacitance (mF)**	0.5	0.35 ± 0.02	2.7 ± 0.1	14.0 ± 2.5
	1	0.27 ± 0.04	1.9 ± 0.3	12.2 ± 1.9
	5	0.22 ± 0.02	1.0 ± 0.2	10.3 ± 1.0
	10	0.19 ± 0.03	0.9 ± 0.1	8.6 ± 0.5

Table S3: **EIS Fitting Parameters from Figure S16**.

**Printed Structure**	R_C (kΩ)	R_B (kΩ)	CPE$_1$ Q_0	CPE$_1$ α	C_B (pF)	CPE$_2$ Q_0	CPE$_2$ α	R_I (kΩ)	$C_{\text{eff},1}$ (μF/cm^2)	$C_{\text{eff},2}$ (μF/cm^2)
**pn Comb (+1 V)**	46.3 ± 6.9	10.6 ± 6.4	1.8 ± 0.9 x 10^{-5}	0.32 ± 0.08	51.7 ± 65.7	9.4 ± 0.1 x 10^{-8}	0.92 ± 0.01	3.1 ± 0.1 x 10^{4}	21.3	4.13
**ppnn Comb (+1 V)**	115 ± 2	9.9 ± 1.6	5.3 ± 0.1 x 10^{-5}	0.71 ± 0.01	152 ± 56	2.6 ± 0.2 x 10^{-8}	0.85 ± 0.01	58.2 ± 0.6	1630	0.33

Note: Fitted parameters are defined with respect to the equivalent circuit in Figure S16c. C_{eff} is calculated from the Brug model, where $C_{\text{eff},1} = Q_0^{1/\alpha} R_B^{1/\alpha-1}$ and $C_{\text{eff},2} = Q_0^{1/\alpha} R_I^{1/\alpha-1}$. CPE circuit elements have been denoted as C_1 and C_2 rather than C_{EDL} and C_{IDL}, respectively, because of the high degree of uncertainty with the equivalent circuit fit.

Table S4: **Stability of Rectification Ratio in Literature**.

**Ref. #**	**Electrolyte Materials**	**Electrode Material**	**Device Area (cm^2)**	**RR**	**Time in Operation (min)**	$\frac{RR_f}{RR_i}$	C_{eff} ($\mu F/cm^2$)
1	Poly(ionic liquid) and neutral gel	Conductive carbon paper	1	11	NR	NR	0.02
15	Ionogels	FTO Glass	1	24	17	56%	1.4
16	Poly(ionic liquid)	Microporous carbon electrodes	1	550	1	NR	0.7
20	Hydrogel	Platinum	4	9.9	NR	NR	48.3
24	Poly(ionic liquid)	ITO-coated PET electrodes	1	23.5	NR	NR	20
25	Poly(ionic liquid)	Microporous carbon electrodes	0.25	14	1	100%	2.1
29	Hydrogel	Platinum	0.196	40	10	NR	NR
32	Hydrogel	Platinum	1	158.6	25	91%	NR
33	Hydrogel/ Hydrogel solution	Ag/AgCl	7.5×10^{-5}	NR	NR	NR	NR
37	Hydrogels	Microporous carbon electrodes	0.24	123	NR	NR	6.7
38	Hydrogel	Platinum	1.9×10^{-5}	215	200	57%	NR
39	Hydrogel	PEDOT:PSS	0.008	5	NR	NR	NR
41	Poly(ionic liquid)	Ag/eGaIn	1	46	2	108%	NR
44	Hydrogel	Platinum	1	10	10	85%	NR
45	Hydrogel	Platinum	0.50	70	5	67%	NR
46	Hydrogel	ITO-coated PET electrodes	4	6	10	83%	NR
52	Poly(ionic liquid)	eGaIn	1	22	NR	30%	0.9
61	Poly(ionic liquid)	eGaIn	0.25	25.4	200	88%	NR
This work	Poly(ionic liquid)	ITO	0.025	46	200	14%	2.1
This work	Poly(ionic liquid)	Acetylene black	0.025	4	200	25%	3.6×10^3

Note: Max Rectification Ratio = RR; RR_f = Final RR; RR_i = Initial RR; C_{eff} = Effective capacitance; NR = Not reported.

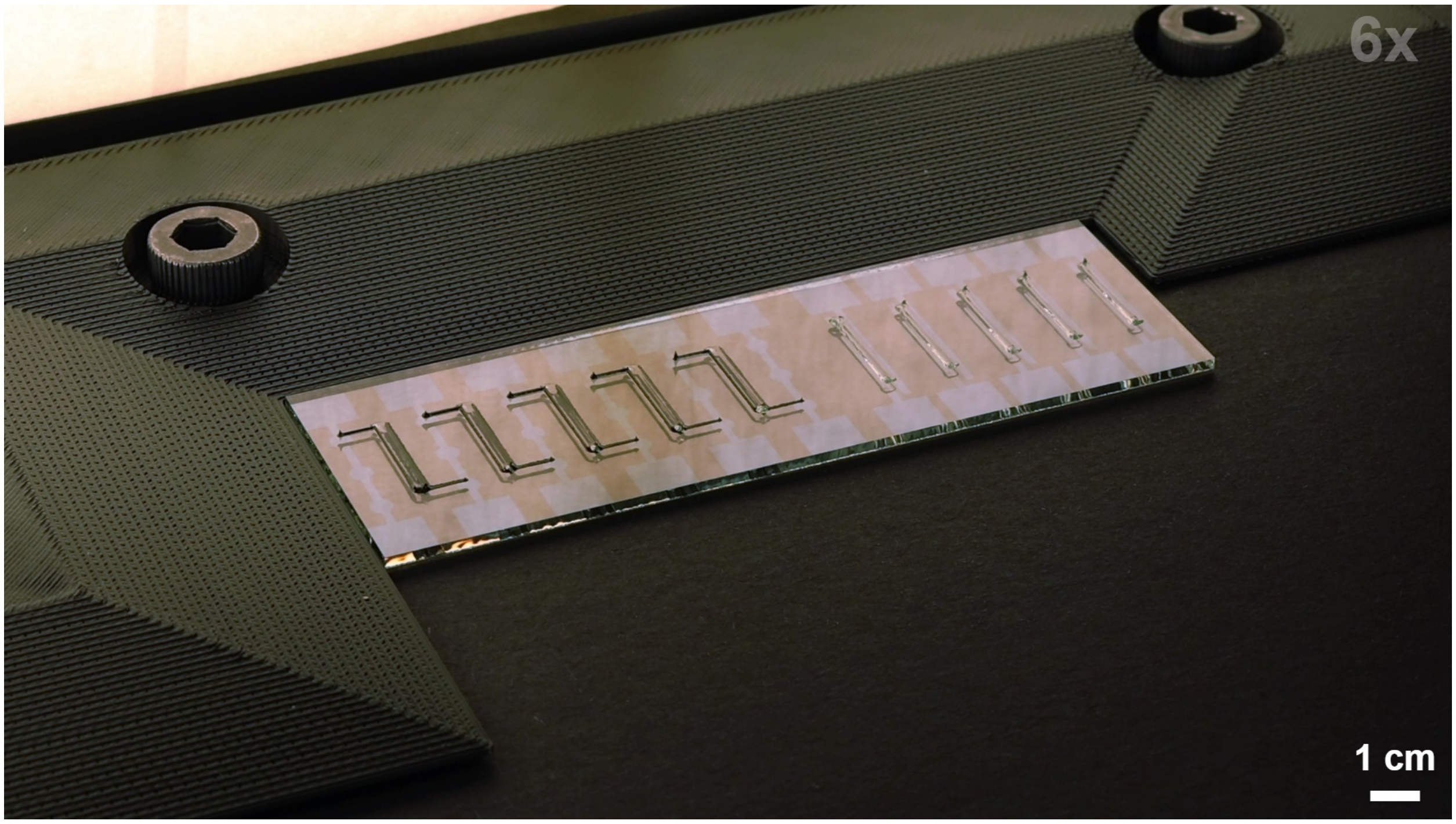

Video S1: **Multimaterial DIW of Printed Comb Diodes.** The video shows the print process for five ppnn and pn comb diodes printed on custom-patterned ITO glass substrates. All inks are extruded through 200 μm nozzles. Electrolyte inks are UV cured for 15 seconds before switching to the next material. Video playback is 6x.
